# Adoptive T cell transfer and host antigen-presenting cell recruitment with cryogel scaffolds promotes long-term protection against solid tumors

**DOI:** 10.1038/s41467-023-39330-7

**Published:** 2023-06-15

**Authors:** Kwasi Adu-Berchie, Joshua M. Brockman, Yutong Liu, Tania W. To, David K. Y. Zhang, Alexander J. Najibi, Yoav Binenbaum, Alexander Stafford, Nikolaos Dimitrakakis, Miguel C. Sobral, Maxence O. Dellacherie, David J. Mooney

**Affiliations:** 1 John A. Paulson School of Engineering and Applied Sciences, Harvard University, Cambridge, MA USA; 2 The Wyss Institute for Biologically Inspired Engineering Harvard University, Boston, MA USA

**Keywords:** Biomaterials, Immunotherapy

## Abstract

Although adoptive T cell therapy provides the T cell pool needed for immediate tumor debulking, the infused T cells generally have a narrow repertoire for antigen recognition and limited ability for long-term protection. Here, we present a hydrogel that locally delivers adoptively transferred T cells to the tumor site while recruiting and activating host antigen-presenting cells with GMCSF or FLT3L and CpG, respectively. T cells alone loaded into these localized cell depots provided significantly better control of subcutaneous B16-F10 tumors than T cells delivered through direct peritumoral injection or intravenous infusion. T cell delivery combined with biomaterial-driven accumulation and activation of host immune cells prolonged the activation of the delivered T cells, minimized host T cell exhaustion, and enabled long-term tumor control. These findings highlight how this integrated approach provide both immediate tumor debulking and long-term protection against solid tumors, including against tumor antigen escape.

## Abstract (web summary)

Adoptive T cell therapy has shown remarkable promise for the treatment of haematological malignancies. Here, the authors show that a cryogel platform for the simultaneous delivery of autologous T cells and recruitment of local antigen-presenting cells promotes long-lasting responses against solid tumours in mice.

## Introduction

Adoptive T cell therapy involves the ex vivo manipulation of T cells and their subsequent reinfusion into patients^1^. The T cells are either genetically modified autologous polyclonal T cells isolated from apheresed PBMCs, or tumor-infiltrating lymphocytes (TILs), which are harvested from tumors, expanded ex vivo and reinfused into patients^2^. Adoptive T-cell therapy has shown remarkable promise in hematological cancers but has seen limited efficacy in solid tumors^3^. Inspired by reports that localized, intra-tumoral injection of T cells yields superior therapeutic outcomes^4,5^, recent efforts have focused on developing biomaterial-based T cell depots which can be implanted or injected at the tumor site to further enhance therapeutic T cell efficacy^6–10^. These T cell reservoirs have taken the form of either pre-formed macroporous scaffolds that can be implanted at the tumor site^6–8^, or injectable in-situ forming gels^9,10^, and are typically fabricated to release T cell bioactive factors like IL-2 or IL-15^6–10^. While adoptively transferred T cells are sufficient for immediate tumor debulking, their long-term efficacy can be hindered by their narrow antigen receptor repertoire, which can result in tumor antigen escape, T cell exhaustion and limited long-term protection^11–13^. An early report however points to improved therapeutic outcomes in mice when biomaterial-based localized delivery of T cells is combined with innate immune regulators^7^.

**Fig. 1:**
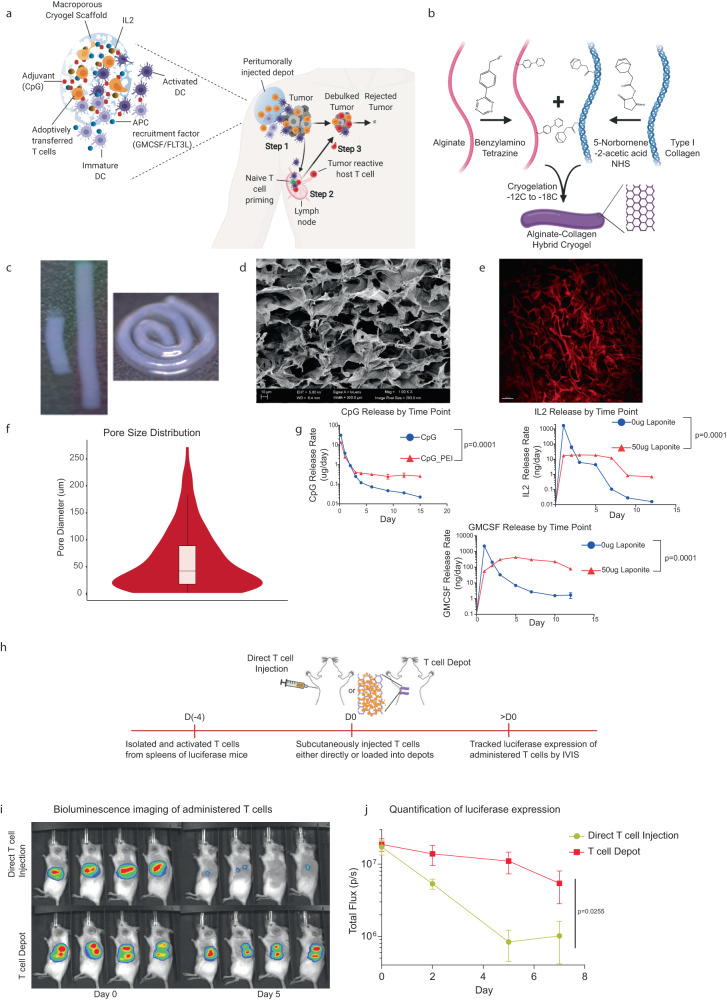
Synthesizing and characterizing SIVETs. **a** Schematic detailing the proposed mechanism of action. **Step 1:** Depots infused with T cells and loaded with factors that recruit antigen-presenting cells (APC) are injected adjacent to a tumor. Depots concurrently release T cells to debulk the tumor while recruiting APCs to the tumor site, where they become activated and process tumor antigen. **Step 2:** Tumor-antigen presenting APCs migrate to lymph nodes and prime host naïve, antigen-specific T cells. **Step 3:** Primed tumor-reactive host T cells traffic to the tumor, which is undergoing tumor debulking by the adoptively transferred T cells, and facilitate tumor rejection and long-term anti-tumor immunity. **b** Schematic of SIVET fabrication. Alginate and collagen type 1 are modified with tetrazine and norbornene respectively, and then reacted under cryogelation conditions (−12C to −18C) to form macroporous alginate-collagen hybrid cryogels. **c** Photographs of rod-shaped cryogels showing tunable lengths (left) and flexibility (right). **d** SEM image of cryogel showing macroporous structure. Image is representative of 8 SEM images from two cryogels. **e** SHG demonstrating pristine cryogel architecture and pore-size distribution. **f** Quantification of pore-size distribution from SHG represented as a violin plot. Data show the distribution of 150 pores. Boxplot information: minima = 1.6, maxima = 271.6, lower bound = 1.6, upper bound = 182.9, 25th percentile = 18.04, center = 42.19, 75th percentile = 88.83. **g** Tunable release of immunomodulatory factors from cryogels: CpG with or without PEI condensation (top left), IL2 and GM-CSF with or without pre-adsorption onto laponite (top right and bottom respectively). *p*-values for **g**–**i** was determined by two-way ANOVA with repeated measures. Data are mean ± s.d. from *n* = 3 for CpG and GMCSF, and *n* = 2 for IL2. **h**–**j** Analysis of local T cell persistence in vivo. **h** Schematic of the experimental set-up. CD8+ T cells were isolated from spleens of luciferase-expressing mice, activated in vitro, and either directly injected subcutaneously or loaded into depots before injection. **i** Bioluminescence images of administered T cells over time. **j** Quantification of T cell luciferase expression over time. *p*-value was determined by two-way ANOVA with repeated measures. Data are mean ± s.d. from *n* = 4.

In this study, we hypothesized that a system that would simultaneously enhance adoptive T cell therapy and engage host immunity might result in effective tumor debulking and provide long-term protection against solid tumors. To address this hypothesis, we developed a biomaterial platform, referred to as synergistic in situ vaccination enhanced T cell depot (SIVET), to locally deliver adoptively transferred T cells to the tumor site while recruiting and activating host antigen-presenting cells (APCs) to process and present antigens from the dying tumor cells to promote anti-tumor host T cell responses (Fig. 1a). It was found that the therapeutic efficacy of T cells alone loaded into the localized depot was enhanced compared with systemic delivery and direct peritumoral T cell delivery. Locally delivering T cells while concentrating APCs via the release of either FLT3L or GM-CSF from the hydrogels elicited host T cell responses and resulted in long-term tumor control in most mice. Phenotypic analysis of immune cells at both the depot and tumor site showed crosstalk between the adoptively transferred T cells and host immune cells. This approach also provided enhanced protection against tumor antigen escape.

## Results

### Synthesizing and characterizing SIVETs

Synergistic In-situ Vaccination Enhanced T cell depot (SIVETs) were fabricated by modifying alginate and type I collagen with tetrazine and norbornene respectively, which react bio-orthogonally via inverse electron demand Diels–Alder click reaction^14^. Under cryogelation conditions, this reaction yields macroporous cryogels with shape recovery properties that readily enable needle injection (Fig. 1b). The collagen provides ligands for T cell adhesion and motility^15^ while the alginate provides structural support to the depot. The depots are rod-shaped, which enhanced scalability, as the length of the depots could be easily tuned (Fig. 1c). The depots are also highly flexible, allowing them to conform to the area of injection (Fig. 1c). Scanning electron microscopy and second harmonic imaging confirmed that the depots contained pores with an average size of 50 µm, with collagen comprising the pore walls (Fig. 1d–f). Changing the ratio of alginate to collagen significantly altered the ability of the depots to recover their shape after deformation, while the total percentage of polymer influenced their interconnected porosity (Supplementary Fig. 1).

To assess the ability of SIVETs to release soluble immunomodulatory factors, the cytokines IL2 and GMCSF, which facilitate T cell expansion and myeloid cell recruitment respectively^16,17^, and the adjuvant CpG, which activates myeloid cells^18^, were loaded into depots by adding the factors to the gel mixture before cryogelation. The interactions of these bioactive factors with the depot were altered either by adsorption onto highly negatively charged laponite nanoparticles (for cytokines)^19^ or by polyethyleneimine condensation (for CpG)^20^ prior to loading (Supplementary Fig. 2a), resulting in tunable release profiles for all three factors (Fig. 1g, Supplementary Fig. 2b–d). Immunomodulatory factors were loaded into depots in concert with laponite or PEI for subsequent studies. Importantly, while negligible cytokine levels were detected in circulation when GMCSF-loaded depots were subcutaneously injected into mice (Supplementary Fig. 2e), GMCSF remained detectable in the depots 7 days after injection (Supplementary Fig. 2f).

To establish the need for adhesion motifs, T cells were subsequently loaded into either alginate-only cryogels or alginate–collagen hybrid depots and their motility analyzed. T cells loaded into alginate–collagen hybrid depots showed significantly faster motility speed and longer track lengths than T cells loaded into alginate-only cryogels (Supplementary Fig. 3a–d, Supplementary Movies 1, 2).

When the loading capacity of the depots was assessed by loading increasing numbers of T cells into the depots and determining the number of T cells that were actually loaded, it was observed that about 10^7^ T cells could be loaded with negligible cell loss, beyond which the loaded T cell numbers plateaued (Supplementary Fig. 3e). For subsequent in vivo experiments, 2 × 10^6^ T cells were loaded into each depot, with two depots being injected per mouse.

To assess the effects of the depots on T cell phenotype, T cells were cultured for 4 and 7 days in the depots in vitro and profiled using flow cytometry. Analyses of relative CD4 and CD8 levels showed that T cells in depots had more balanced CD4–CD8 ratios by day 7 while 2D controls were more skewed towards CD8 (Supplementary Fig. 4a, c). Importantly, CD4+ FOXP3+ levels remained low for both 2D and depot conditions (Supplementary Fig. 4b, d). Unsupervised phenotypic analyses of T cells revealed the presence of multiple T cell populations (Supplementary Fig. 4e–h). Further analyses showed that while T cells in both depots and 2D culture showed similar activation levels at day 4, T cells in depots maintained higher levels of activation than T cells cultured in 2D at day 7, being more enriched in CD25 + LAG3 + PD1 + OX40+ clusters (clusters 2–5) (Supplementary Fig. 4i–k).

After confirming that the depots do not lead to increased FOXP3+ levels in vitro, we sought to assess whether IL2 release from depots could induce host T cell FOXP3+ generation in vivo. Subcutaneous injection of either blank or IL2-loaded depots showed low levels of CD4+ FOXP3+ T cells in the scaffolds after 7 days of injection (Supplementary Fig. 5).

### T cell depots enhance local T cell persistence in vivo and provide superior tumor control

Local in vivo T cell persistence following administration of cell-loaded depots was next investigated. CD8+ T cells from the spleens of luciferase-expressing mice^21,22^ were activated and either directly injected subcutaneously into mice with soluble IL2 (Direct T cell Injection) or infused into T cell depots with matched IL2 quantity before injection (T cell Depot). IVIS images and subsequent quantification showed that while similar amounts of T cells were delivered on Day 0, there were significantly more luciferase-expressing CD8+ T cells at the site of injection at subsequent timepoints with T cell depots (Fig. 1h–j). A similar trend was observed when T cell-loaded depots were injected into the intraperitoneal (IP) space of mice (Supplementary Fig. 6).

We next investigated if T cells loaded into depots enter into circulation after subcutaneous injection. CD8+ T cells isolated from pmel mice, which recognize the gp100 antigen on B16-F10 tumor cells^23,24^ were first loaded into depots and administered subcutaneously into mice. Analysis of blood samples 9 days after T cell delivery showed that pmel T cells could be detected in circulation (Supplementary Fig. 7).

**Fig. 2:**
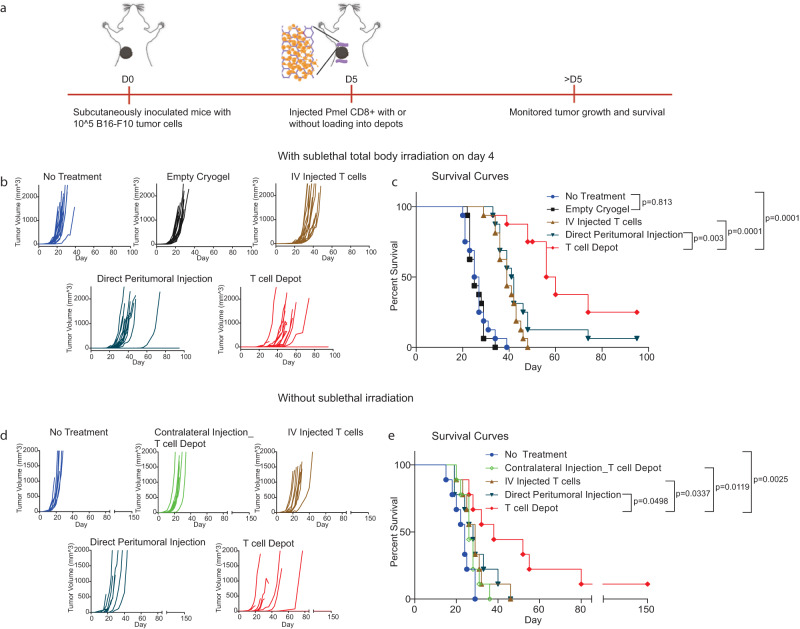
T cell depots alone enhance B16-F10 tumor control. **a** Schematic of therapeutic study. **b**, **c** Tumor volumes (**b**) and Kaplan–Meier survival curves (**c**), with sub-lethal irradiation preconditioning of mice left untreated, injected peritumorally with empty depots, or treated with the following T cell conditions: intravenous T cell delivery, direct peritumoral T cell injection or T cell depots. *P*-values for **c** were determined using a log-rank (Mantel–Cox) test. Data are representative of *n* = 16 mice per condition in two independent experiments (8 mice per group per experiment). **d**, **e** Therapeutic study without preconditioning. Tumor growth (**d**) and Kaplan–Meier survival curves (**e**) of the indicated conditions. For the ‘Contralateral Injection_T cell Depot’ condition, T cell-loaded depots were injected contralateral to the tumor location. *P*-values for **e** were determined by Log-rank (Mantel–Cox) test. Data represent *n* = 9 mice per condition for a single experiment.

The therapeutic efficacy of cells administered via T cell depots was subsequently examined in the aggressive B16-F10 tumor model after preconditioning by sub-lethal irradiation, a common clinical procedure to improve T cell engraftment in adoptive therapies^25^. CD8+ T cells isolated from pmel mice were administered to tumor-bearing mice 5 days after B16-F10 inoculation when tumors had become established (Fig. 2a, Supplementary Fig. 8). For preconditioning, mice were subjected to total body sub-lethal irradiation on day 4 after tumor inoculation. Tumor growth and subsequent mouse survival showed that peritumoral injection of T cell depots infused with pmel CD8+ T cells resulted in significant enhancement of tumor control over intravenously (IV) injected T cells and direct peritumoral T cell injection (Fig. 2b, c). Importantly, the peritumoral injection of empty depots did not affect tumor growth.

Next, the ability of T cell-only depots to control B16-F10 tumors without sub-lethal irradiation was investigated, to assess the ability of T cell depots to enable transferred T cell engraftment without prior preconditioning. T cells administered by IV or direct peritumoral injection showed significantly diminished tumor control when pre-conditioning was not performed. However, T cell-only depots still showed superior control of B16-F10 tumors (Fig. 2d, e). The therapeutic effect was significantly reduced when the T cell depots were injected contralateral to the tumor site (Fig. 2d, e), confirming the significance of localized T cell delivery. Subsequent studies were performed without sublethal irradiation.

### SIVETs elicit host T-cell responses

**Fig. 3:**
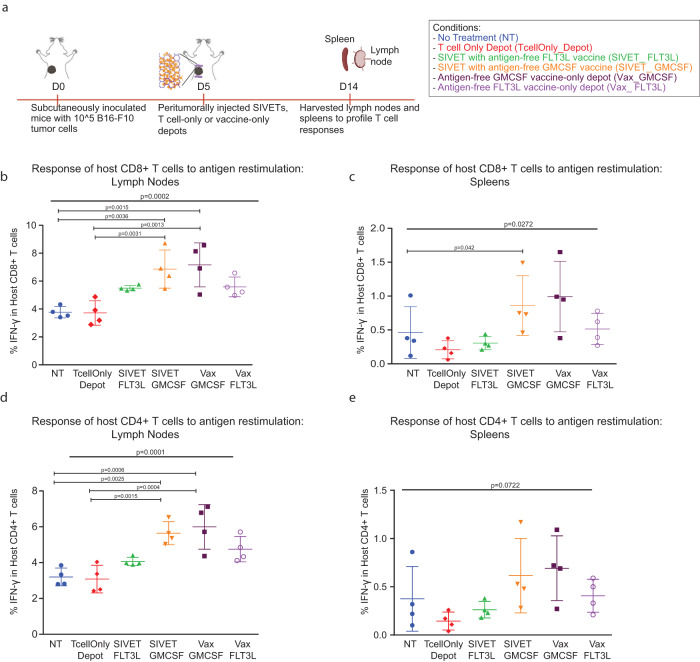
SIVETs elicit host T-cell responses. **a** Schematic of the experiment. The following conditions were investigated: (1) no treatment controls (NT), (2) T cell only depots (TcellOnly_Depot), (3) SIVET with antigen-free FLT3L vaccine (SIVET_ FLT3L), (4) SIVET with antigen-free GMCSF vaccine (SIVET_ GMCSF), (5) antigen-free GMCSF vaccine-only depot (Vax_GMCSF) and (6) antigen-free FLT3L vaccine-only depot (Vax_ FLT3L). **b**, **c** Proportions of IFN-γ-expressing host CD8+ T cells isolated from lymph nodes (**b**) and spleens (**c**) after in vitro antigen re-stimulation. **d**, **e** Proportions of IFN-γ-expressing host CD4+ isolated from lymph nodes (**d**) and spleens (**e**) after in vitro antigen re-stimulation. *P*-values were determined using two-tailed one-way ANOVA with Geisser–Greenhouse correction. Pairwise *P*-values were determined by performing Tukey. Adjusted *P*-values < 0.05 are shown. Data are mean ± s.d. from *n* = 4 mice per condition for a single experiment.

SIVET both locally delivers T cells and concentrates and activates host antigen-presenting cells (APCs) to the tumor site for antigen uptake and both local antigen presentation and migration to lymph nodes for host T cell priming. After establishing that T cell depots by themselves resulted in better therapeutic outcomes compared with traditional modes of T cell delivery, we sought to understand the impact of the complete SIVET on both adoptively transferred T cells and host immune cells. The relative fractions of tumor-reactive host T cells were first analyzed in lymph nodes and spleens 9 days after treating B16-F10 tumor-bearing mice with one of the following: (1) no treatment controls (NT), (2) T cell only depots (TcellOnly_Depot), (3) SIVET with antigen-free FLT3L vaccine (SIVET_ FLT3L), (4) SIVET with antigen-free GMCSF vaccine (SIVET_ GMCSF), (5) antigen-free GM-CSF vaccine-only depot (Vax_GMCSF) and (6) antigen-free FLT3L vaccine-only depot (Vax_ FLT3L) (Fig. 3a). FMS-like tyrosine kinase 3 ligand (FLT3L)^26^ and Granulocyte-macrophage colony-stimulating factor (GM-CSF)^16^ were used to recruit antigen-presenting cells (APCs), while the TLR9 agonist, class B CpG, was included in all the SIVET and vaccine-only conditions to activate recruited APCs^18^. The vaccines were formulated without antigens, as dying cells from the tumors could serve as antigen sources for the peritumorally injected depots. Functional analyses revealed the presence of tumor-reactive host T cells in lymph nodes and spleens after antigen re-stimulation in vitro, with SIVET and vaccine-only conditions showing higher percentages of IFN-γ expressing host CD4+ and CD8+ T cells (Fig. 3b–e). The proportions of tumor-reactive T cells present in the lymph nodes and spleens were higher in the GM-CSF than in FLT3L conditions.

### H&E staining of both depots and tumor sections reveals treatment-dependent cellular profiles

Hematoxylin and eosin (H&E) staining of both depots and tumor sections was next performed. Significantly higher cellularity was seen at the site of the SIVET_GMCSF depots relative to the other conditions, with many cells located at the interface between the depot and the native tissue, suggesting that the SIVETs do not trap the recruited cell populations. Importantly, there was markedly higher infiltration of immune cells at the tumor site for the SIVET-treated conditions compared to the NT control, signaling a more pronounced immune activity in the former tumors (Supplementary Fig. 9).

### SIVETs enhance the relative levels of activated antigen-presenting cells in depots and tumors

**Fig. 4:**
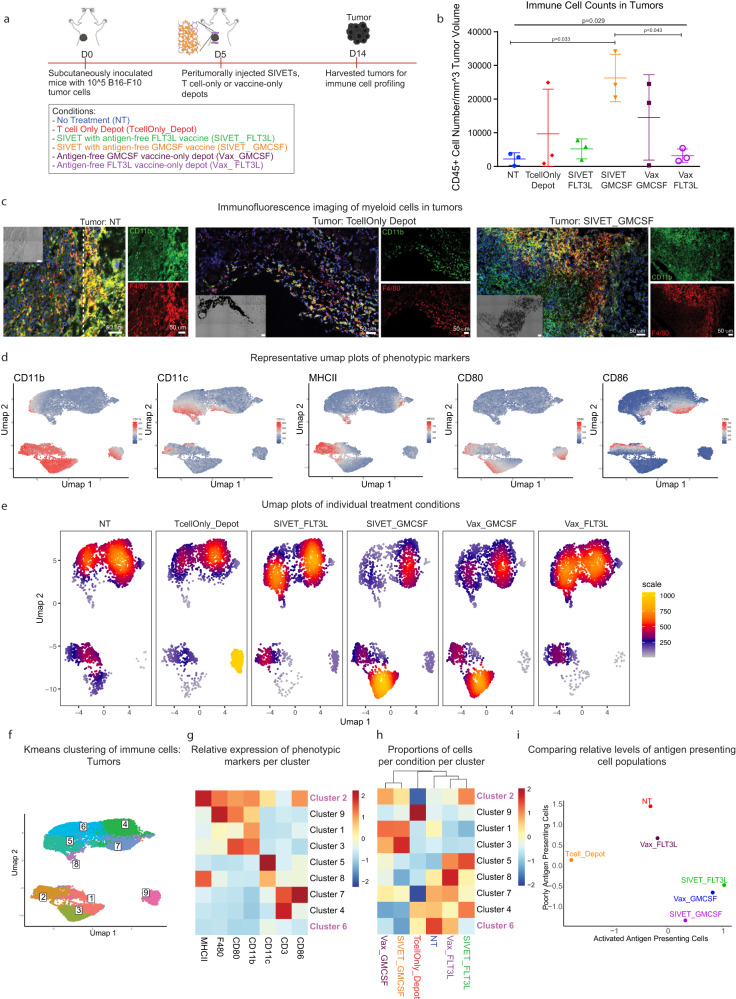
SIVETs enhance the relative levels of activated antigen-presenting cells in tumors. **a** Schematic of the experiment. **b** Total numbers of tumor-infiltrating CD45+ cells per mm^3^ of tumor volume. *P*-value was determined by two-tailed one-way ANOVA with Geisser–Greenhouse correction. Pairwise *P*-values were determined by performing Tukey. Adjusted *P*-values < 0.05 are shown. Data are mean ± s.d. from *n* = 3 mice per condition for a single experiment. **c** Immunofluorescence imaging of myeloid cells expressing the indicated markers in NT, TcellOnly_Depot and SIVET_GMCSF tumors. Images are tiled acquisitions from a slide selected from 30 continuous sections each from 1 mouse per condition. **d** Umap plots showing the expression of the indicated markers. **e** Umap density plots of individual treatment conditions showing distinct localization of cells based on treatment group. Denser (hot) regions indicate more cells. **f** Umap plot overlaid with Kmeans clusters of immune cells in tumors. **g** Heatmap plot showing the average expression of the indicated immune cell markers in each cluster after *K*-means analysis. **h** Heatmap plot showing the proportion of cells in each condition represented in each cluster. Some clusters of interest are highlighted. **i** Scatterplot comparing the relative enrichment levels of the activated antigen-presenting cell population (cluster 2), and the poorly antigen-presenting cell population (cluster 6) as a function of the treatment group. Data are pooled cells from *n* = 3 mice per condition for a single experiment.

Broad phenotypic profiling of immune cells in both the tumors and depots was next performed to further understand the crosstalk between the adoptively transferred T cells and host immune cells (Fig. 4a, Ext. Fig. 10a). The total numbers of immune cells infiltrating the tumors were influenced by their respective treatment condition, with SIVET_GMCSF having the highest levels of tumor-infiltrating immune cells (Fig. 4b). Accordingly, immunofluorescence imaging of myeloid cells in the tumors revealed a markedly high infiltration of myeloid cells in the SIVET_GMCSF condition compared with the TcellOnly_Depot and NT conditions (Fig. 4c). Umap and Kmeans analyses also showed distinct immune cell populations within the tumor (Fig. 4d–h). Cluster 2, which comprises myeloid cells expressing canonical markers of activation and antigen presentation was enriched in the SIVET and vaccine-only conditions (Fig. 4f, g). A CD11c-mid, CD11b-low, MHCII-low, CD80-low, and CD86-low population (cluster 6), likely a non-activated myeloid population with poor antigen presentation, was most present in the NT control, with notable levels in the TcellOnly_Depot condition. Importantly, the SIVET conditions had lower proportions of cells in this non-activated myeloid population than the TcellOnly_Depot condition and their respective vaccine-only conditions (Fig. 4g, h). Thus, SIVETs resulted in an enhancement of myeloid cells expressing markers of activation and antigen presentation and a concomitant reduction in non-activated myeloid populations with poor antigen presentation ability (Fig. 4i).

The phenotypic profiles of immune cells in the depots mirrored those in the tumors. The total numbers of immune cells infiltrating the depots were dependent on treatment condition, with SIVET_GMCSF and Vax_GMCSF recruiting significantly more cells than the other conditions (Supplementary Fig. 10b). Umap analyses of immune cells in the depots showed the presence of both T cell and myeloid cell populations (Supplementary Fig. 10c). Immune cells in the various conditions were phenotypically distinct and localized to different regions on the umap plot (Supplementary Fig. 10d). Cluster 5, which comprises a myeloid cell population expressing canonical markers of activation and antigen presentation, was most enriched in SIVET_ FLT3L and Vax_ FLT3L and least enriched in the TcellOnly_Depot condition (Supplementary Fig. 10e–g). Importantly, the proportions of cells present in cluster 5 were higher in the SIVET conditions than their corresponding vaccine-only conditions, irrespective of the APC recruitment factor used (Supplementary Fig. 10e–g). Additionally, when normalized for immune cell number, the proportions of the activated antigen-presenting cells in cluster 5 were highest in the SIVET_GMCSF condition and remained higher in the SIVETs than their corresponding vaccine-only conditions (Supplementary Fig. 10h).

### SIVETs minimize host T cell exhaustion in tumors and prolong activation of adoptively transferred T cells in depots

**Fig. 5:**
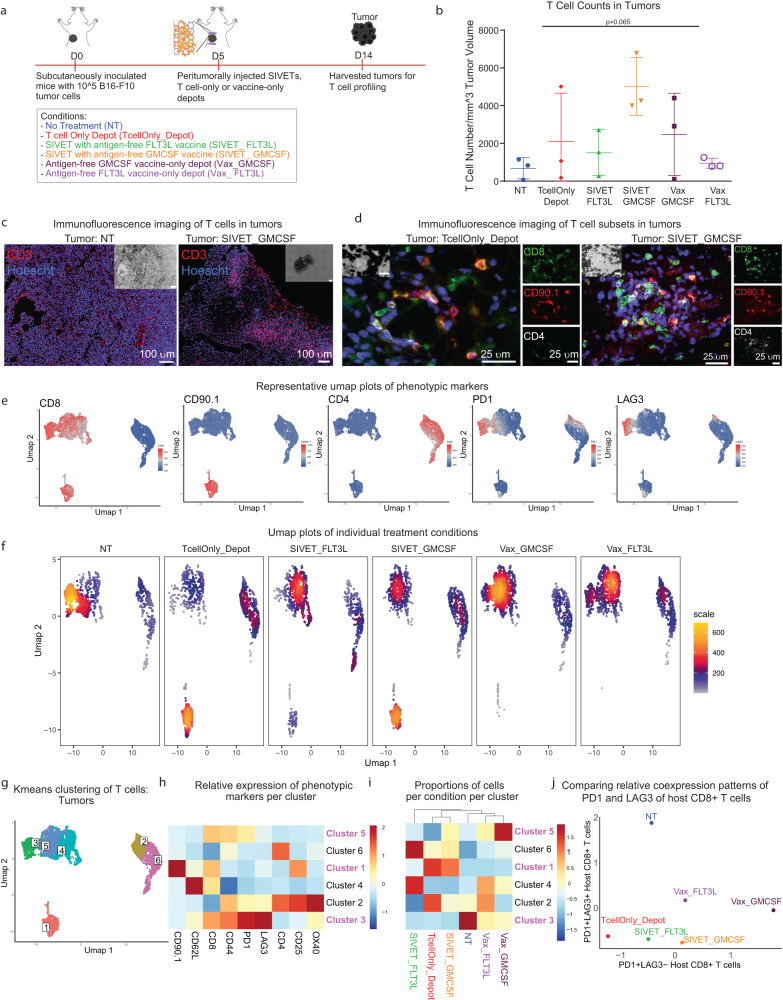
SIVETs minimize host T cell exhaustion in tumors. **a** Schematic of the experiment. **b** Numbers of tumor-infiltrating T cells per mm^3^ of tumor volume. *P*-value was determined by two-tailed one-way ANOVA with Geisser–Greenhouse correction. Data are mean ± s.d. from *n* = 3 mice per condition for a single experiment. **c**, **d** Immunofluorescence imaging of T cells present in NT and SIVET_GMCSF (**c**), as well as specific T cell subtypes in TcellOnly_Depot and SIVET_GMCSF tumors (**d**). Images are tiled acquisitions from a slide selected from 30 continuous sections each from 1 mouse per condition. **e** Umap plots showing the expression of the indicated markers. **f** Umap density plots of individual treatment conditions showing distinct localization of cells based on treatment group. Denser (hot) regions indicate more cells. **g** Umap plot overlaid with Kmeans clusters of T cells in tumors. **h** Heatmap plot showing the average expression of the indicated T cell markers in each cluster after *K*-means analysis. **i** Heatmap plot showing the proportion of cells in each condition represented in each cluster. Some clusters of interest are highlighted. **j** Scatterplot comparing the relative enrichment levels of PD1+ LAG3− host CD8+ T cells (cluster 5), and PD1+ LAG3+ host CD8+ T cells (cluster 3) as a function of the treatment group. Data are pooled cells from *n* = 3 mice per condition for a single experiment.

T cell-specific profiles in tumors and depots were subsequently assessed (Fig. 5a, Ext. Fig. 11a). T cell numbers infiltrating the tumors varied by treatment condition, with SIVET_GMCSF having the highest levels (Fig. 5b). SIVET conditions had higher numbers of tumor-infiltrating T cells than their respective vaccine-only conditions (Fig. 5b). Consequently, immunofluorescence imaging of T cells in tumors showed a noticeably higher T cell presence in the SIVET_GMCSF tumors relative to the NT control (Fig. 5c). Both adoptively transferred, and host T cells were present in tumors of TcellOnly_Depot and SIVET_GMCSF treated groups (Fig. 5d). Umap and Kmeans analyses of T cells in tumors also revealed distinct localization of T cell populations (Fig. 5e, f). The adoptively transferred T cell population (cluster 1) was enriched in the TcellOnly_Depot condition tumors, while the SIVET conditions had higher proportions of host T cells (Fig. 5e–i). Phenotypic analyses of host T cells in the tumors revealed a presumably exhausted CD8+ population expressing high levels of LAG3 and PD1 (cluster 3) (Fig. 5e–i). Both umap and kmeans analyses showed that while this presumably exhausted T cell cluster (cluster 3) was mostly enriched in the NT control, the vaccine-only conditions had notable proportions of these T cells, which were dramatically diminished in their corresponding SIVET conditions (Fig. 5e–i). A second PD1 expressing, LAG3-low host T cell cluster (cluster 5), which likely comprises non-exhausted T cells that are actively engaging tumor cells, was notably present in the SIVET conditions with a higher enrichment relative to the TcellOnly_Depot (Fig. 5e–i). SIVETs, therefore, resulted in T cell populations that actively engaged tumor cells while minimizing their exhaustion (Fig. 5j). The identity of the CD4+ CD25+ OX40+ population in cluster 2 is not clear, as these could represent conventional T cells or regulatory T cells within the tumors.

Umap analyses of depots also showed treatment-dependent effects on T-cell profiles. Depots had both CD4+ and CD8+ T cells, with a significant CD90.1+ adoptively transferred T cell population present (Supplementary Fig. 11b, c). The adoptively transferred T cells dominated the TcellOnly_Depot condition while the SIVET conditions had considerable infiltration of host T cells (Supplementary Fig. 11b, c). Kmeans clustering of the adoptively transferred T cells revealed two populations that had a differential expression of CD25 and PD1. The SIVET conditions were enriched in the CD25-hi, PD1-mid population (cluster 5), while the TcellOnly_Depot condition was enriched in the CD25-low, PD1-low cluster (cluster 6) (Supplementary Fig. 11d–f).

### SIVETs result in long-term tumor control

**Fig. 6:**
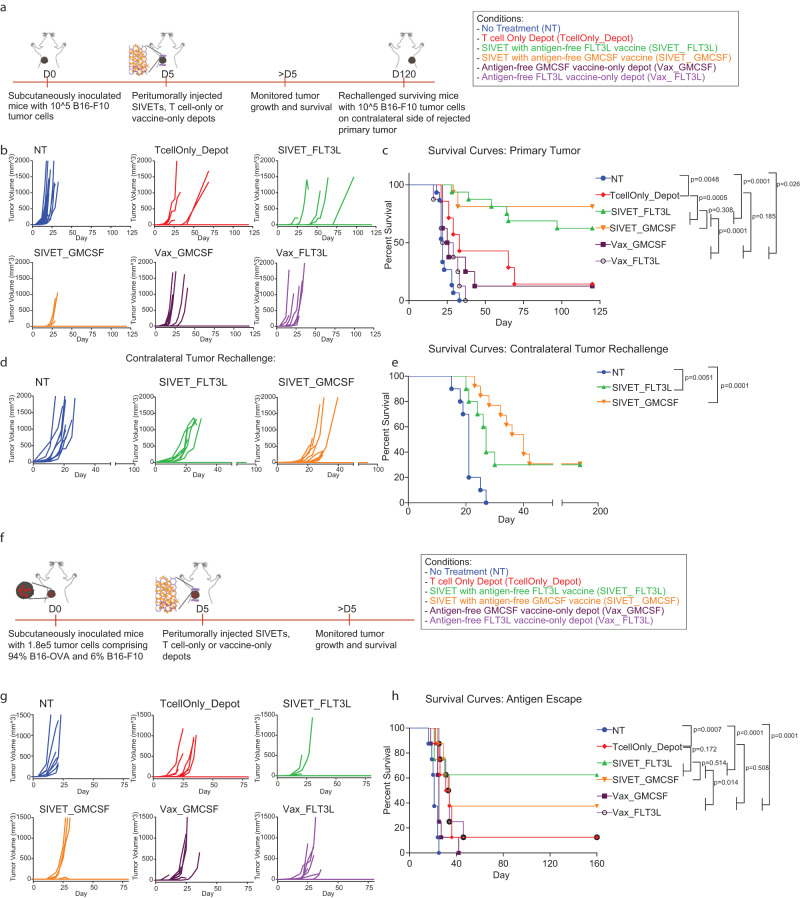
SIVETs enhance long-term tumor control. **a** Schematic of the experiment. **b**, **c** Primary B16-F10 tumor studies. Tumor volumes (**b**) and Kaplan–Meier survival curves (**c**) comparing tumor growth and survival of mice treated with the indicated conditions. *P*-values for **c** were determined by Log-rank (Mantel–Cox) test. Data represent *n* = 7 mice for TcellOnly_Depot condition, *n* = 8 mice for Vax_FLT3L and Vax_GMCSF conditions, and *n* = 15 mice for NT condition in two independent experiments (7 and 8 mice for individual experiments) and *n* = 16 mice for SIVET_FLT3L and SIVET_GMCSF conditions (*n* = 8 mice per experiment). **d**, **e** Contralateral tumor re-challenge studies for long-term surviving mice. **d** Tumor growth (**d**) and Kaplan-Meier survival curves (**e**) of mice contralaterally re-challenged with 1 × 10^5^ B16-F10 tumor cells after 120 days of primary tumor challenge. p-values for e were determined by Log-rank (Mantel–Cox) test. Data are representative of *n* = 10–13 mice per condition in two independent studies. **f**–**h** Antigen escape study. **f** Schematic of therapeutic study for (**g**, **h**). Tumor volumes (**g**) and Kaplan–Meier survival curves (**h**) comparing tumor growth and survival of mice treated with the indicated conditions. *P*-values for (**h**) were determined using a log-rank (Mantel–Cox) test. Data represent *n* = 8 mice per condition for a single experiment.

Using the B16-F10 tumor model, we next sought to ascertain whether the observed effects of SIVETs on both adoptively transferred T cell profiles and host immune cells translated into therapeutic benefits. Tumor growth analysis showed that while mice that received either therapeutic TcellOnly_Depot or vaccine-only treatments delayed tumor growth, they eventually succumbed to their tumors. However, mice treated with SIVETs significantly controlled tumor growth, with a majority of these mice completely rejecting their primary tumors. Combined survival analyses from two independent studies showed that 10/16 mice from SIVET_FLT3L and 13/16 mice from SIVET_GMCSF completely rejected their primary tumors long-term (Fig. 6a–c).

Next, long-term surviving mice were re-challenged with the same dose of B16-F10 tumor cells contralateral to the location of the primary tumor after 120 days to assess the ability of memory T cells to mount a systemic immune response against the secondary tumor. Tumor growth and survival analyses showed that long-term surviving mice from the SIVET_FLT3L and SIVET_GMCSF conditions significantly controlled their secondary contralateral tumors, with about a third of the mice in each condition completely rejecting their secondary tumors (Fig. 6d, e). Interestingly, tumors grew slower in the SIVET_GMCSF condition compared with the SIVET_FLT3L condition for the mice that did succumb (Fig. 6d, e).

### SIVET modulates long-term T-cell phenotype

The impact of SIVET on long-term T cell phenotype was next investigated by profiling T cells at 82 days post-tumor inoculation from the lymph nodes and spleens of mice that rejected their primary tumors. Only mice treated with SIVETs were profiled, in addition to naïve mouse controls, as the mice from the other conditions had succumbed to their tumors. Umap and Kmeans analyses of T cells in both lymph nodes and spleens showed that T cell profiles in the SIVET-treated mice were distinct from those of naïve mice (Supplementary Figs. 12, 13). In the lymph nodes, SIVET treatment resulted in an enrichment of more differentiated effector memory populations (CD62L-low, CD44-hi) for both CD8+ and CD4+ compartments (cluster 4 and cluster 8), compared with T cells from the naïve mice, which were mostly enriched in central memory (CD62L-hi, CD44-hi) and naïve (CD62L-hi, CD44-low) T cell clusters (clusters 5 and 6 respectively) (Supplementary Fig. 12b–f). Importantly, umap, Kmeans, and principal component analyses (PCA) revealed that SIVET_GMCSF was enriched in the more differentiated T cell clusters, while SIVET_FLT3L was intermediate (Supplementary Fig. 12g). This trend was maintained in T cells harvested from spleens, where the SIVET_GMCSF condition was again most enriched in the more differentiated T cell clusters (Supplementary Fig. 13).

Next, the long-term phenotype of the adoptively transferred CD90.1+ T cells was assessed. Adoptively transferred T cells were detectable in the lymph nodes and spleens of SIVET-treated mice 82 days after tumor inoculation (Supplementary Fig. 14b, c). Umap and Kmeans analyses of the adoptively transferred T cells from the lymph nodes revealed that the majority of the T cells were central memory (CD62L-hi, CD44-hi) T cells (clusters 1 and 5) (Supplementary Fig. 14d–f). Importantly, there was a notable enrichment of SIVET_GMCSF in cluster 3, which is a more differentiated effector memory (CD62L-low, CD44-hi) T cell population (Supplementary Fig. 14f–h), mirroring the observed phenotypic trends from the host T cell populations.

### SIVET provides enhanced protection against tumor antigen escape

Finally, we tested the ability of the SIVET-induced host T cell response to provide protection against tumor antigen escape by inoculating mice with a mixture of 94% B16-OVA tumor cells and 6% wild-type B16-F10 cells. B16-OVA and B16-F10 share common tumor antigens but do not share the ovalbumin antigen. Mice were subsequently treated with OT1 CD8+ T cells, which recognize the ovalbumin peptide residues 257–264, and monitored for their ability to control both the B16-OVA and B16-F10 tumor cells. Tumor growth and mouse survival analyses showed that while the TcellOnly_Depot and the vaccine-only conditions delayed tumor growth and mouse survival, most of the mice eventually succumbed to the tumors. In contrast, about 60% and 40% of mice treated with either SIVET_FLT3L or SIVET_GMCSF, respectively, completely rejected their tumors long-term (Fig. 6f–h).

## Discussion

Here we addressed the hypothesis that a biomaterial system that simultaneously enhanced adoptive T-cell therapy and provided host immune engagement could provide enhanced immune protection, as evidenced by both immediate tumor debulking and long-term protection against solid tumors. While T cell depots by themselves showed superior therapeutic benefits relative to direct peritumoral T cell injection and IV T cell injection, SIVET resulted in complementary effects between the adoptively transferred T cells and the host immune cells and led to long-term tumor control in most of the mice. Importantly, SIVET also provided enhanced protection against tumor antigen escape.

When used for localized T cell-only delivery, injectable T cell depots resulted in enhanced therapeutic benefits in solid tumors relative to IV and direct peritumoral T cell injection. These observations are consistent with previous studies that have reported greater therapeutic effects of biomaterial-based adoptive T cell delivery relative to traditional modes of T cell delivery^6–10^. While intravenous delivery could be optimal for hematological cancers, T cells delivered this way must traffic to solid tumors, and only a fraction of the adoptively transferred T cells find the tumor at a given time. T cells delivered peritumorally or intratumorally without scaffolds overcome the initial hurdle of trafficking but generally do not persist at the tumor site, likely due to harsh conditions of the tumor microenvironment^27^. Biomaterial-based delivery systems serve as T cell reservoirs that help to overcome the hurdle of trafficking while providing the requisite environment for T cell persistence. Previous biomaterial-based platforms used for adoptive T cell delivery have mostly been in the form of spherical or sheet-like implantable scaffolds^6–8^, or injectable in-situ forming gels^9,10^. Implantable scaffolds require invasive surgeries for administration, and the spherical or sheet-like shapes could pose difficulties in terms of scaling for larger mammals. While injectable, in-situ forming gels may be effective in anatomical locations where an injection pocket could be formed (e.g., subcutaneous space), they likely would have limited applicability in other locations due to dispersion from the injection site before gelation is complete. The injectable, rod-shaped T-cell depot described in this study overcomes these challenges, as it obviates the need for invasive surgery and allows for the use of syringes and catheters. Two depots, which are each 2 mm in diameter and 1 cm were injected. However, the diameter and length of the depots could be easily tuned if desired in future studies. The pre-formed depots could also be injected at multiple anatomical locations while allowing the depots to conform to the area of injection, because of their flexibility.

Phenotypic profiles of the adoptively transferred T cells were influenced by engaging the host immune cells and vice versa. The recruitment and activation of host antigen-presenting cells correlated with greater activation of the adoptively transferred T cells, relative to the T cell-only depot condition. This is consistent with reports that continual help from antigen-presenting cells is important for the anti-tumor efficacy of transferred T cells^28^. Further, it is likely that the reduction in the proportions of non-activated, poorly antigen-presenting myeloid cells in the SIVETs compared with vaccine-only conditions is due to paracrine signaling from the adoptively transferred T cells to the recruited myeloid cells. The dramatic decrease in host CD8+ T cell exhaustion in SIVET-treated tumors relative to the vaccine-only conditions could be due to tumor debulking by the adoptively transferred T cells that reduce antigen density, limiting the over-stimulation of intra-tumoral host T cells. The specific depot agent (GM-CSF vs FLT3L) utilized to concentrate host myeloid cells informed the composition of myeloid cells at the depot and tumor sites and influenced the long-term phenotypic profiles of both the adoptively transferred and host T cells. FLT3L particularly recruited classical antigen-presenting myeloid cell populations, while GMCSF recruited a broader array of myeloid populations and in higher numbers, consistent with the expected behavior of the two cytokines^29,30^. Additionally, the availability of T cell populations with faster effector response, as seen in the higher proportions of long-term effector memory adoptively transferred and host T cell populations for the SIVET_GMCSF condition could explain why tumors grew slower in SIVET_GMCSF than SIVET_FLT3L upon a secondary challenge for mice that succumbed. While we did not observe significant host regulatory T cell induction as a function of IL2 loading in the depots, IL2 has been shown in other studies to potentially lead to regulatory T cell enrichment. Consequently, other cytokines like IL15 could replace IL2 to obviate that concern. Consequently, other cytokines like IL15 could replace IL2 to obviate that concern^31^. The release kinetics of GMCSF was observed to be different in situ compared with in vitro findings, suggesting that it will be important in future studies to fully characterize in vivo release kinetics.

SIVET provided enhanced long-term tumor control and protection against tumor antigen escape, confirming that the phenotypic effects of SIVET on both adoptively transferred T cell profiles and host immune cells translated into long-term therapeutic benefits. The selected ratio of B16-OVA to B16-F10 was empirically chosen, based on the hypothesis that antigen escape begins with a small fraction of cells. Altering these ratios could affect the observed therapeutic outcomes; it is unclear what ratio would best reflect human tumors. Previous studies have shown that approaches that promote resistance of CAR T cells to exhaustion^32^ or directly boost their expansion in vivo^33^ can lead to better therapeutic responses to solid tumors. Enhanced efficacy of adoptive T cell therapy can also be promoted by engaging host immunity with cGAMP^7^. Our study demonstrates the therapeutic benefit of combining adoptive T cell therapy and the active recruitment of antigen-presenting cells for in-situ vaccination, utilizing principles from previous in-situ vaccination work^20,34–38^. The enhanced activation of the adoptively transferred T cells in the SIVET conditions as well as the dramatic decrease in host T cell exhaustion likely allow both adoptively transferred and host T cells to provide enhanced anti-tumor protection individually, and to provide long-term therapeutic benefits against aggressive tumors.

In sum, we have demonstrated a strategy to enhance adoptive T-cell therapy with therapeutic cancer vaccination to achieve long-term anti-tumor efficacy in murine solid tumors, providing a potential path to overcoming some of the clinical limitations of adoptive T-cell therapy in this setting. These findings further motivate the development of additional strategies that can simultaneously provide local and enhanced adoptive T-cell therapy, while eliciting host T-cell responses.

## Methods

### Material synthesis

#### Collagen modification

Rat Tail Collagen Type I (Corning #354236) was modified with 5-Norbornene-2-acetic acid succinimidyl ester (Nb-NHS) (Sigma Aldrich #776173) using a ratio of 1g Nb-NHS: 10 g collagen. First, rat tail collagen was neutralized with NaOH to pH 7.2–7.5, buffered with 10x DPBS, and diluted to an initial concentration of 2 mg/ml. Next, Nb-NHS was dissolved to 2 mg/ml in DMSO and diluted 10x in 1x PBS. An equal volume of Nb-NHS solution was then added to the neutralized collagen under continual stirring, resulting in 1 mg/ml final collagen concentration. The reaction proceeded for 5 h at 4 °C to delay collagen gelation and was quenched with 0.1 N acetic acid to re-acidify the collagen solution. Norbornene-modified collagen (Col-Nb) was then dialyzed against 0.025 N acetic acid for 4 days, filtered through a 0.45-µm filter, and lyophilized.

#### Alginate modification

Alginate tetrazine was synthesized as previously described. Briefly, Pronova ultrapure MVG sodium alginate (Novamatrix) was modified with (4-(1,2,4,5-tetrazin-3-yl)phenyl methanamine hydrochloride (Karebay Biochem) using carbodiimide chemistry. Alginate was first dissolved in 0.1 M 2-(N-morpholino)ethanesulfonic acid (MES), 0.3 M NaCl, pH 6.5 at 5 mg/ml. Next, 1.9 g of ethyl-3-(3-dimethylaminopropyl)-carbodiimide hydrochloride (EDC) (ThermoFisher #22980) and 1.2 g of N-hydroxysuccinimide (NHS) (ThermoFisher #24500) were added per gram of alginate. 0.1 g of tetrazine was then added under constant stirring and allowed to react overnight at room temperature. The tetrazine-modified alginate was subsequently purified first by tangential flow filtration (KrosFlow KR2i; Spectrum Labs) against a 150 to 0 mM decreasing NaCl gradient with a 1 kDa MWCO membrane, and then by treatment with 1 g activated charcoal. This was followed by filtration through a 0.22-µm filter and subsequent lyophilization.

### Fabrication of depots

To fabricate 0.75 wt%/vol hybrid alginate–collagen cryogel depots comprising 60% alginate and 40% collagen, lyophilized Col-Nb was first dissolved to 9 mg/ml in 0.025 N acetic acid at 4 °C for at least 48 h. Alginate tetrazine was freshly dissolved on the day of cryogel fabrication to 2% wt/vol and cooled at 4 °C. Dissolved Col-Nb was neutralized with cold 1 N NaOH, buffered with cold 10x DPBS and balanced with cold milliQ water, after which the alginate tetrazine was added, yielding 3 mg/ml and 4.5 mg/ml final concentrations of collagen and alginate, respectively. The cryogel mixture was immediately pipetted into a 2 mm diameter Tygon tubing (VWR #89404-318) at 50 µl cryogel mixture per 1 cm tubing, and placed in a −15 °C freezer overnight for cryo-polymerization. After cryogelation, cryogels were thawed at room temperature and ejected by gently flushing the tygon tubing with 400 µl DPBS. Depots with different ratios of alginate to collagen or total polymer content were fabricated by varying the relative final concentrations of the alginate or collagen (while keeping the total polymer amount constant), or by increasing the total amount of polymer respectively.

### Estimation of cryogel interconnected porosity and shape recovery

Cryogel interconnected porosity and shape recovery were estimated as previously described. Briefly, Cryogel interconnected porosity was investigated by first weighing intact cryogels (*M*_hydrated_), wicking cryogels with kimwipe for 15 s to remove the excess buffer from cryogel pores and re-weighing the wicked cryogels (*M*_wicked_). Interconnected porosity was quantified as follows:

$$\frac{{M}_{{{\rm {hydrated}}}}-{M}_{{{\rm {wicked}}}}}{{M}_{{{\rm {hydrated}}}}}$$

Equation (1): Estimation of interconnected porosity

Cryogel shape recovery/memory was estimated by first weighing intact cryogels (*M*_hydrated_), wicking cryogels with kimwipe for 15 s to remove excess buffer, rehydrating the gels in DPBS and re-weighing the rehydrated cryogels (*M*_rehydrated_). Shape recovery was quantified as follows:

$$\frac{{M}_{{{\rm {rehydrated}}}}}{{M}_{{{\rm {hydrated}}}}}$$

Equation (2): Estimation of cryogel shape recovery

### SEM and SHG characterization of depots

#### SEM

Cryogels were fixed with 2.5% glutaraldehyde and 2% PFA in the presence of 0.115 M sucrose and 100 mM Hepes for 1 h. Scaffolds were then washed in 100 mM Hepes and 0.115 M sucrose 3 times, each for 5 min, followed by 1% osmium staining for 1 h and 1% Uranyl acetate staining overnight. After staining, samples were washed 3 times with milliQ water and serially dehydrated with 50%, 70%, 95% and 100% ethanol for 10 min each. Scaffolds were then freeze fractured in liquid nitrogen, dried using critical point drying, mounted onto stubs, coated with platinum/palladium for 10 s, and imaged with the Ultra plus FESEM at 3 keV with gun vacuum at 2.29 × 10^−10^ mbar and system vacuum at 1.43 × 10^−6^ mbar. Images were taken using the secondary electron (SE2) detector.

#### SHG

Pristine cryogels were imaged via second harmonic generation using a Leica SP5 X MP Inverted Confocal Microscope at 820 nm wavelength at 10x magnification. Pore size distribution was determined using Imaris.

### Incorporation of bioactive factors into depots

Bioactive factors like IL2, GMCSF, FLT3L, and CpG were incorporated into cryogels by adding them to the gel mixture before cryogelation. To achieve controlled release, recombinant mouse IL2 (Biolegend #575408) and recombinant murine GM-CSF (Peprotech #315-03) or recombinant murine FLT3L (Peprotech #250-31L) were preloaded onto charged laponite XLG (BYK additives) as follows. First, laponite was dissolved at 30 mg/ml in milliQ with extensive vortexing until the solution became clear. For cryogels loaded with both IL2 and GM-CSF/FLT3L, 5 µg of IL2/50 µl gel solution and 1 µg of GM-CSF or FLT3L/50 µl gel solution were each separately adsorbed onto 25 µg laponite for 1 h at 4 °C. For cryogels loaded with IL2 only, 5 µg of IL2/50 µl gel solution was adsorbed onto 50 µg laponite for 1 h at 4 °C. This kept the total amount of laponite in the scaffold at 50 µg. 50 µg of CpG ODN 1826 (Invivogen #tlrl-1826-1) was adsorbed onto 3 µg linear 25 K PEI (Polysciences 23966) for 1 h at 4 °C. The preloaded bioactive factors were then added to the gel mix before the gels underwent cryogelation as described above.

CpG release studies were performed using the Quant-iT OliGreen ssDNA Kit (Thermo #O11492), while cytokine release was quantified via ELISA.

### T cell isolation, activation, culturing

Splenic mouse CD8+ T cells were isolated with magnetic-bead-based CD8α+ T Cell Isolation Kit, mouse (Miltenyi #130-104-075) following the manufacturer’s protocol. T cells were activated with Dynabeads® Mouse T-Activator CD3/CD28 (ThermoFisher Scientific # 11452D) at a 1:1 ratio in T cell media: RPMI 1640 (Lonza #BE12-702F), 10% heat-inactivated fetal bovine serum (Gibco #10-082-147), 1% pen/strep, 55 μM *β*-mercaptoethanol, 10 mM HEPES (Sigma Aldrich # H4034), 1% 100x non-essential amino acid (Lonza #13-144E), 100 mM sodium pyruvate (Lonza #13-115E), supplemented with recombinant mouse IL-2 (BioLegend #575404).

### T cell motility assay

To perform the T cell motility assay, T cells were first stained with 0.5 µM cell tracker deep red (ThermoFisher #C34565) and loaded into pre-formed cryogels by briefly wicking the scaffolds using kimwipe and rehydrating them in 25 µl of 2 × 10^5^ cells/ml concentrated T cell solution. Time-lapse imaging was taken at ×20 magnification for 2 h at 60-s intervals and analyzed using Imaris.

### Animal studies

Animal studies were approved by the National Institutes of Health and the Harvard University Faculty of Arts and Sciences Institutional Animal Care and Use Committee (IACUC).

#### In vivo T cell persistence study

To investigate local in vivo T cell persistence following administration of T cell-loaded depots, CD8+ T cells were first isolated from the spleens of luciferase-expressing female FVB-Tg(CAG-luc,-GFP)L2G85Chco/J mice (Jackson #008450) and activated for 4 days as described above, after which the dynabeads were removed. Depots loaded with 5 µg recombinant murine IL2 adsorbed onto 50 µg laponite were infused with 2 × 10^6^ luciferase expressing CD8+ T cells. Two depots per mouse were subcutaneously injected into the left flank of female C57 albino mice (Jackson #000058). 4 × 10^6^ luciferase expressing CD8+ T cells with matched IL2 were directly injected subcutaneously to serve as controls. Luciferase expression was tracked using bioluminescence imaging by subcutaneously administering 150 mg/kg of d-luciferin (GoldBio #LUCK-100) and measuring luminescence via IVIS (Perkin Elmer)

#### B16-F10 therapeutic tumor studies

##### Tumor inoculation

1 × 10^5^ B16-F10 tumor cells at passage 7 (ATCC #CRL-6475) were subcutaneously injected into the left flank of 7–9 weeks old C57BL/6J mice (Jackson #000664) on day 0 and treated on day 5 (refer to source data for sex of mice for the individual studies) when the tumors had become palpable. For studies with sublethal irradiation preconditioning, mice were subjected to 5 Gy γ-irradiation on day 4. *T cell isolation and loading into depots:* CD8+ T cells were isolated from the spleens of B6.Cg-Thy1a/Cy Tg(TcraTcrb)8Rest/J (pmel) mice (Jackson #005023), which recognize the gp100 antigen on B16-F10 tumor cells, and activated using dynabeads for 4 days. The activated T cells were then separated from the dynabeads and infused into depots at 2 × 10^6^ T cells/cryogel. *Depot composition:* Depots were fabricated as detailed above. Depots designated for T cell delivery only were loaded with 5 µg of recombinant murine IL2 adsorbed onto 50 µg laponite. SIVETSs had 5 µg recombinant murine IL2 and 1 µg recombinant murine GMCSF or FLT3L individually adsorbed onto 25 µg laponite, as well as 50 µg CpG ODN 1826 adsorbed onto PEI. Vaccine-only depots received the same formulation but without T-cell infusion. *Injection of depots:* Two depots were injected per mouse, peritumorally on either side of the tumor in 100 µl DPBS using a 16-gauge needle. For IV T cell injection and direct peritumoral injection, 4 × 10^6^ pmel T cells with matched IL2 were administered either by tail vein injection or injected subcutaneously next to the tumor in 100 µl DPBS using a 25-gauge needle. *Tumor volume measurements and mice survival:* For tumor volume determination, calipers were used to measure the tumor length, width and height. Tumor volume was estimated as 0.5*length*width*height. Where the tumor was flat, a width of 0.5 mm was assumed. In accordance with Harvard IACUC guidelines, mice were euthanized when tumors grew to 20 mm in any dimension, when tumors became necrotic, or when excessive weight loss was observed in tumor-bearing mice.

#### Antigen escape tumor model

1.8 × 10^5^ tumor cells comprising 94% B16-cOVA (a kind gift from the Wucherpfennig lab) and 6% B16-F10, both at passage 7 were subcutaneously injected into the left flank of 8 weeks old C57BL/6J mice. CD8+ T cells were isolated from the spleens of female C57BL/6-Tg(TcraTcrb)1100Mjb/J (OTI) mice (Jackson #003831), which recognize the ovalbumin peptide residues 257–264, and activated using dynabeads for 4 days. T cells were separated from the dynabeads and infused into the depots as described above. Depot composition, tumor volume measurements, and criteria for mice survival are the same as previously described.

### Tissue processing

#### Tumors

Tumors were excised into gentleMacs C tubes (Miltenyi #130-093-237) containing 150U/ml collagenase type IV (Thermo #17104019) and 0.1 µg/µl DNAse 1 (Sigma #11284932001) in digestion media: RPMI 1640 + 10% FBS. Tumors were mechanically dissociated using the gentleMacs tissue dissociator (Miltenyi #130-093-235) program m_spleen_03, and incubated for 25 min at 37 °C. Tumors were then mechanically dissociated for the second time using the same program and incubated for 15 min. The enzymatic reaction was quenched using MACS buffer: DPBS with 0.5% BSA and 2 mM EDTA, and filtered through a 30-µm strainer (Miltenyi #130-098-458).

#### Lymph nodes

Brachial, axillary, and inguinal lymph nodes were harvested and pooled before digestion. The same digestion protocol highlighted above was used to digest the lymph nodes.

#### Depots

Depots were harvested into gentleMacs C tubes containing 150U/ml collagenase type IV, 0.1 µg/µl DNAse 1 and 2 U/ml alginate lyase (Sigma #A1603). The same digestion protocol described above was used to digest depots.

#### Spleens

Harvested spleens were mechanically dissociated by using 1 ml syringe plungers to mash the spleens through 30-µm strainers. Red blood cell lysis (BioLegend #420302) was performed on a single cell suspension for 1 min before further downstream processing.

### In vitro T cell restimulation

Lymph nodes and spleens were first digested as described above. To perform in vitro T cell stimulation, lymph node and spleen-derived cells were incubated with a cocktail of 2 µg/ml mgp100, M27, and M30 peptides in addition to 5 × 10^4^ B16-F10 tumor cells. The broad approach to antigen stimulation was taken because the vaccine was antigen-free, and thus a broad repertoire of T cell clones was expected to be elicited by the vaccines. After 1.5 h, 0.27 µl of the GolgiStop protein transport inhibitor (BD #554724) was added to each well, after which the cells were incubated for 4 h. The cells were then processed for flow cytometry.

### Flow cytometry

#### Surface staining

Cells were kept at 4 °C throughout immunostaining. First, cells were stained with LIVE/DEAD™ Fixable Blue Dead Cell Stain (ThermoFisher Scientific #L23105) at 1000x dilution for 30 min in PBS, after which staining was quenched with flow cytometry staining (FACs) buffer (Invitrogen #00-4222-26). Cells were blocked with TruStain FcX Fc receptor blocking solution (BioLegend #101319) for 5 min and stained with surface protein antibodies for 20 min (1:50 dilution), after which the cells were washed 3x in FACs buffer. Flow cytometry acquisition was performed on a BD Fortessa LSRII. Single color compensation beads (Thermo #01-2222-41), were used for multi-parameter flow cytometry compensation. Gating was done based on fluorescence-minus-one (FMO) controls. The complete set of antibodies used for flow cytometry is listed in Supplementary Table 3.

#### Intracellular cytokine staining (ICS) and FOXP3 Staining

ICS was performed after live/dead and surface staining, using the Cyto-Fast™ Fix/Perm Buffer Set (BioLegend #426803) according to the manufacturer’s protocol. Briefly, cells were fixed in the Cyto-Fast™ Fix/Perm Buffer for 20 min at room temperature, washed twice in 1X Cyto-Fast™ Perm Wash solution and stained in 1X Cyto-Fast™ Perm Wash solution for 20 min (1:50 dilution) at room temperature. After staining, cells were washed 3× in FACs buffer before acquisition on the BD Fortessa LSRII. For FOXP3 staining, cells were fixed and permeabilized using the True-Nuclear™ Transcription Factor Buffer Set (BioLegend #424401) according to the manufacturer’s protocol, after which FOXP3 was stained for 30 min at room temperature. Cells were then washed and acquired on the BD Fortessa LSRII.

### Flow cytometry analyses

#### Flowjo analyses

Fcs files exported from the BD Fortessa LSRII cytometer were imported into Flowjo, analyzed using the following hierarchy: SSC-A/FSC-A to gate for lymphocytes → FSC-H/FSC-A to gate for single cells → CD3/Live_Dead to gate for live T cells (or CD45/Live_Dead for total immune cells) → CD4/CD8 to gate for CD4+ or CD8+ T cells. Further downstream gating is performed using FMO controls, or compensated single-cell flow cytometry intensity values are exported as csv files for unsupervised analyses. Sample gating strategy is shown in Supplementary Fig. 15.

#### Unsupervised analyses

##### Umap analyses

Exported single cell flow cytometry intensities were imported into R version 4.0.5 and pooled together. Outlier intensities, defined as values outside 3 standard deviations from the mean (in both directions), were discarded. Cells were then down-sampled to keep the same number of cells per condition, after which umap analyses were performed. Umap projections were plotted as 2D scatter plots using ggplot2 version 3.3.3^1^, and annotated with either the marker intensity or condition. Umaps of the individual conditions were plotted either as 2D scatter plots or as 2D scatter heatmaps (LSD R package 4.1.0^2^) by subsetting the umap projections of the condition of interest from the projection of the pooled dataset. *Kmeans*: Kmeans was performed by first discarding outlier intensities as described above. To estimate the appropriate number of clusters (K), and Elbow plot was generated by estimating the total within sum of squares iterated over *K* = 1 to *K* = 20. The optimum *K* was chosen as the Elbow point on the plot, where increasing K does not lead to substantial changes in the total within sum of squares. Using the optimum number of clusters, Kmeans clustering was performed on the cells, after which the cluster each cell belongs to was overlaid onto the independently generated umap plot to show concordance. To determine the phenotypic markers that characterize a specific cluster, the cluster centers, which represent the mean expression of each marker in each cluster were estimated. The proportions of cells per condition per cluster were also determined and were represented as heatmaps using the Pheatmap package version 1.0.12^3^. *Principal component analysis (PCA)*: PCA was performed on the frequencies of each condition in each cluster to assess the similarity between the different conditions. PCA scores and loadings for the first two principal components were then plotted using the factoextra package version 1.0.74 in R.

### Immunofluorescence imaging

Tumors were excised and fixed for 1 h in 4% PFA at 4 °C. Tissues were cryopreserved in 30% sucrose overnight and then frozen in OCT solution. Tissues were then cryo-sectioned into 20–30 µm slices and mounted on superfrost plus slides. For immunostaining, the OCT was removed by immersing the sections in 1×PBS, blocked with 5% normal rat serum, 5% normal mouse serum, and purified rat anti-mouse CD16/CD32 for 30 min. Finally, samples were stained using the manufacturer’s specifications. Slides were washed 2× with 1×PBS, stained with Hoescht 33342, mounted in ProLong Gold Antifade Mountant (Thermo Fisher) and covered with a no. 1.5 coverslip (Electron microscopy services). Tissues were imaged via tiled acquisitions on an LSM 710 confocal microscope using a 32x water immersion objective. Images were stitched and processed in the Zen Black software. The complete set of antibodies used for immunofluorescence imaging is listed in Supplementary Table 3.

### H&E

Tissue sections were manually stained by H&E. Briefly, sections were hydrated using decreasing concentrations of ethanol and then stained by hematoxylin (Mayer’s hemalum solution, Sigma, USA). Differentiation and bluing agents (Differentiation solution and Scott’s Water, both from Sigma, USA) were then used to augment hematoxylin staining. Eosin stain (Eosin Y solution, Sigma USA) was then applied, followed by ethanol dehydration. Imaging was done on an Echo Revolve Microscope.

### Statistical analyses

Unless otherwise specified, all statistical analyses were performed on Prism Graphpad software version 9.0.2. Statistical tests used Student’s t-test with Welch’s correction for comparison between two groups and two-tail one-way ANOVA with Geisser–Greenhouse correction for group comparisons. Two-way ANOVA with repeated measures was used for the release of study datasets and the Log-rank (Mantel–Cox) test for mouse survival studies. *P*-value < 0.05 was considered significant unless otherwise noted. Error bars represent standard error of mean unless otherwise noted. Detailed statistical information is provided in Supplementary Tables 1 and 2.

### Reporting summary

Further information on research design is available in the Nature Portfolio Reporting Summary linked to this article.

## Supplementary information

Supplementary Information

Supplementary movie 1

Supplementary movie 2

Reporting Summary

## Data Availability

Raw or analyzed data will be made available upon request. Source data are provided with this paper.

## Source data

Source Data
