# Supplementary material for: Adoptive T cell transfer and host antigen-presenting cell recruitment with cryogel scaffolds promotes long-term protection against solid tumors
Source: Nat Commun. 2023 Jun 15;14:3546. doi: 10.1038/s41467-023-39330-7 (PMC10272124; doi:10.1038/s41467-023-39330-7)
Supplement: Supplementary file 1 — Supplementary Information [file 41467_2023_39330_MOESM1_ESM.pdf]

Supplementary Information

Adoptive T cell transfer and host antigen-presenting cell recruitment with cryogel scaffolds promotes long-term protection against solid tumors

Kwasi Adu-Berchie[1,2], Joshua M. Brockman[1,2+], Yutong Liu[1,2+], Tania W. To[2], David K.Y. Zhang[1,2], Alexander J. Najibi[1,2], Yoav Binenbaum[2], Alexander Stafford[2], Nikolaos Dimitrakakis[2], Miguel C. Sobral[1,2], Maxence O. Dellacherie[1,2], David J. Mooney[1,2*]

[1]John A. Paulson School of Engineering and Applied Sciences, Harvard University, Cambridge, Massachusetts, USA.
[2]The Wyss Institute for Biologically Inspired Engineering Harvard University, Boston, Massachusetts, USA.

[+] Authors contributed equally

*Correspondence: David J. Mooney (mooneyd@seas.harvard.edu)

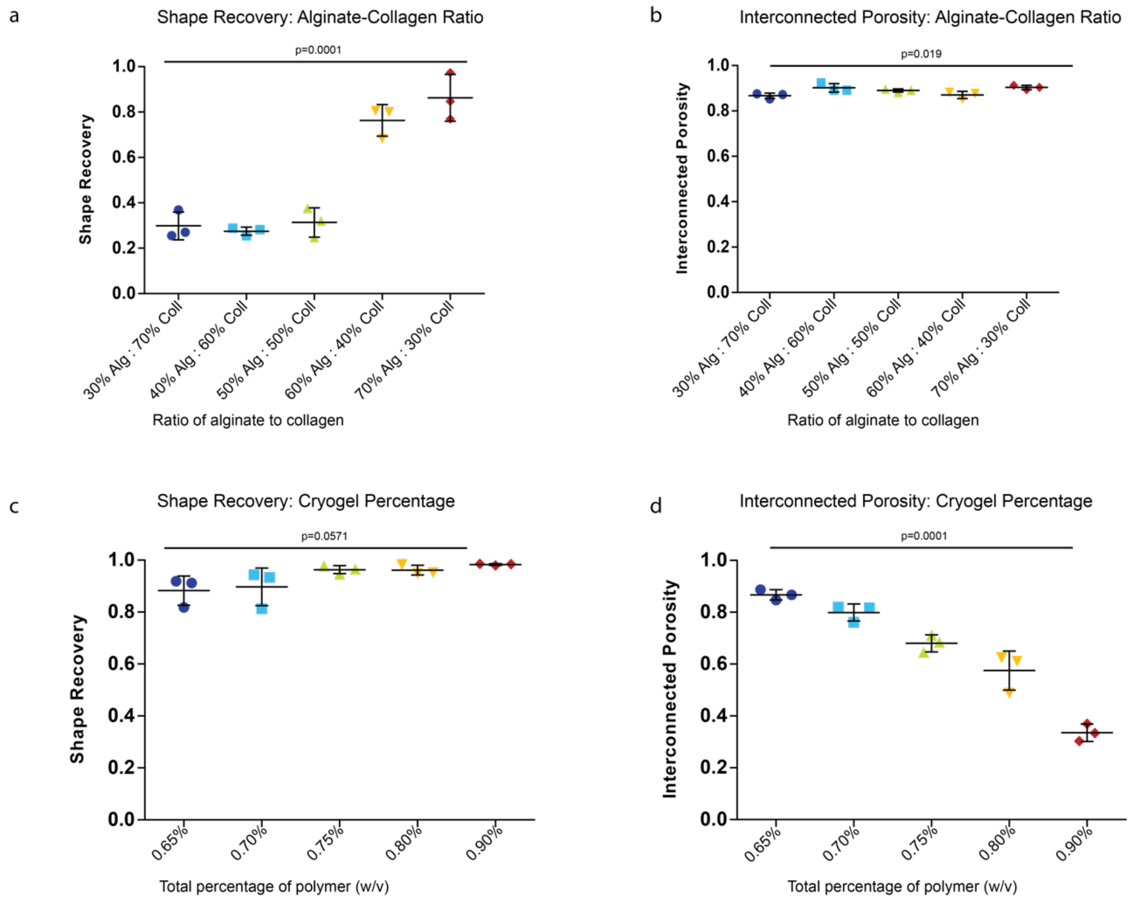

Supplementary Fig. 1 Further characterization of SIVETs. a-b. Cryogel shape recovery and interconnected porosity profiles as a function of changing the ratios of alginate to collagen. c-d. Same profiles as in a-b as a function of varying total percentage of polymer. P-values determined by two-tailed one-way ANOVA with Geisser-Greenhouse correction. Data represent mean ± s.d. from n=3.

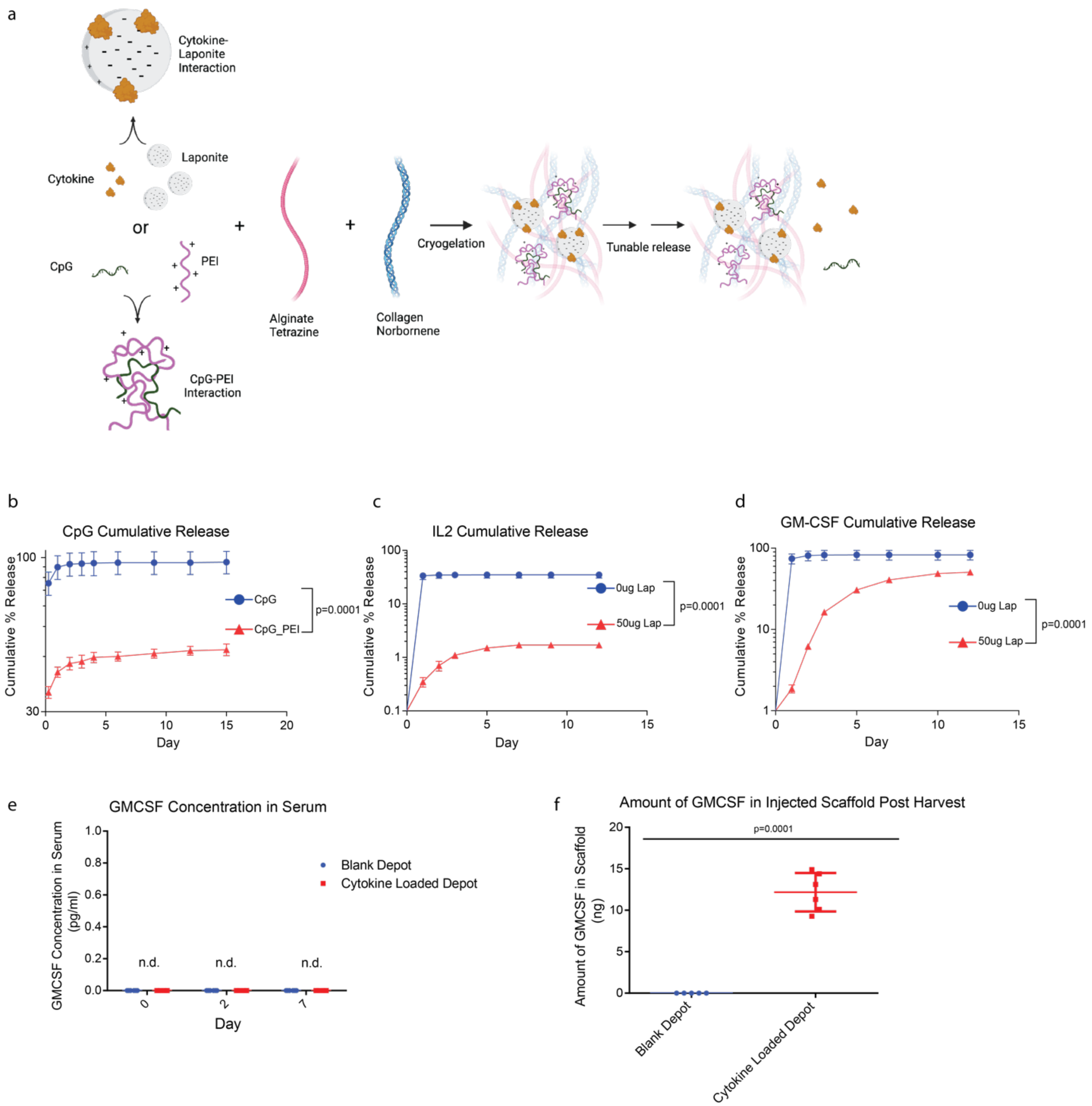

Supplementary Fig. 2 Further characterization of bioactive factor release from depots. a. Schematic of tunable release of bioactive factors from depots. To achieve tunable release of bioactive factors, cytokines (eg. IL2, GMCSF) and adjuvants (CpG) were either directly added to cryogel mixture before cryogelation, or pre-adsorbed onto laponite (for cytokines) or PEI (for CpG) before they were added to cryogel solution. b-d. Cumulative release of immunomodulatory factors from cryogels: b. CpG with or without PEI condensation, c. IL2 and d. GM-CSF with or without pre-adsorption onto laponite. P-values were determined by two-way ANOVA with repeated measures. Data are mean ± s.d. from n=3. e. Levels of GMCSF detected in serum of mice at the indicated days after subcutaneous administration of either blank depot or GMCSF loaded depot. n.d.=not detectable. f. Amount of GMCSF detected in depots 7 days after subcutaneous administration. Two depots were injected per mice, each with 1ug GMCSF. P-values was determined by performing two-tailed unpaired t-test with Welch's correction. Data are mean ± s.d. from n=5 mice for blank depot and n=6 mice for GMCSF loaded depot.

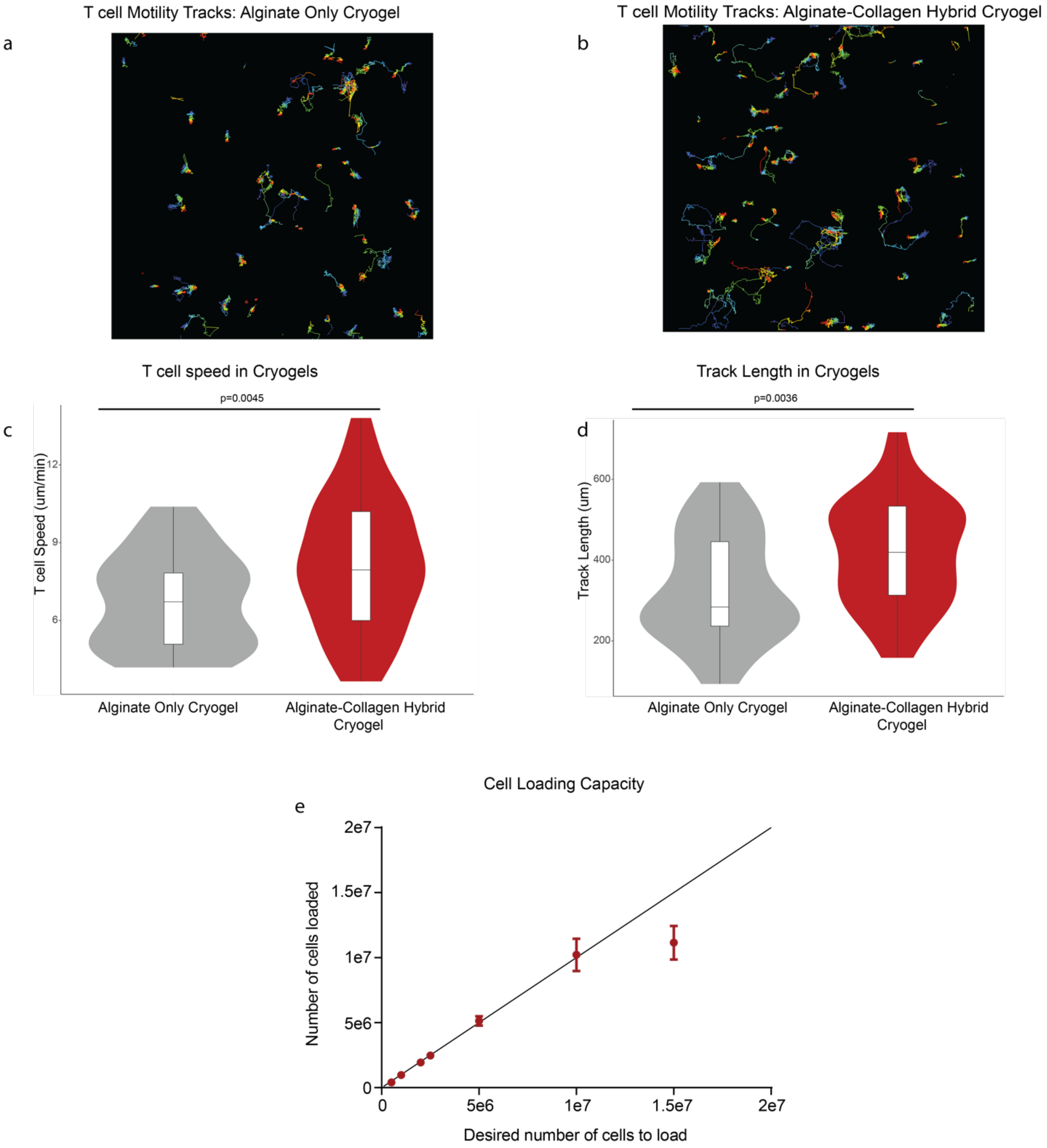

Supplementary Fig. 3 Characterizing T cell motility and loading capacity in depots. a-b. Motility tracks for T cells loaded in alginate-only cryogels (a) or alginate-collagen hybrid depots (b). c-d. Violin plots comparing T cells speeds (c) and track lengths (d) for T cells loaded in alginate and alginate-collagen hybrid depots. P-values determined by performing two-tailed unpaired t test with Welch's correction. Boxplot data speed and track lengths of 40 T cells. Boxplot information for 1) T cell speed for Alginate Only Cryogel: minima=4.2, maxima=10.38, lower bound=4.2, upper bound=10.38, 25th percentile=5.08, center=6.72, 75th percentile=7.83, 2) T cell speed for Alginate-Collagen Hybrid Cryogel: minima=3.66 maxima=13.8, lower bound=3.66, upper bound=13.8, 25th percentile=6, center=7.95, 75th percentile=10.2, 3) Track length for Alginate Only Cryogel: minima=93.8, maxima=592, lower bound=93.8, upper bound=592, 25th percentile=236, center=284, 75th percentile=446, 4) Track length for Alginate-Collagen Hybrid Cryogel: minima=159, maxima=716, lower bound=159, upper bound=716, 25th

percentile=313, center=419, 75th percentile=534. e. Plot showing T cell loading in depots for increasing numbers of T cells loaded. Data are mean ± s.d. from n=4.

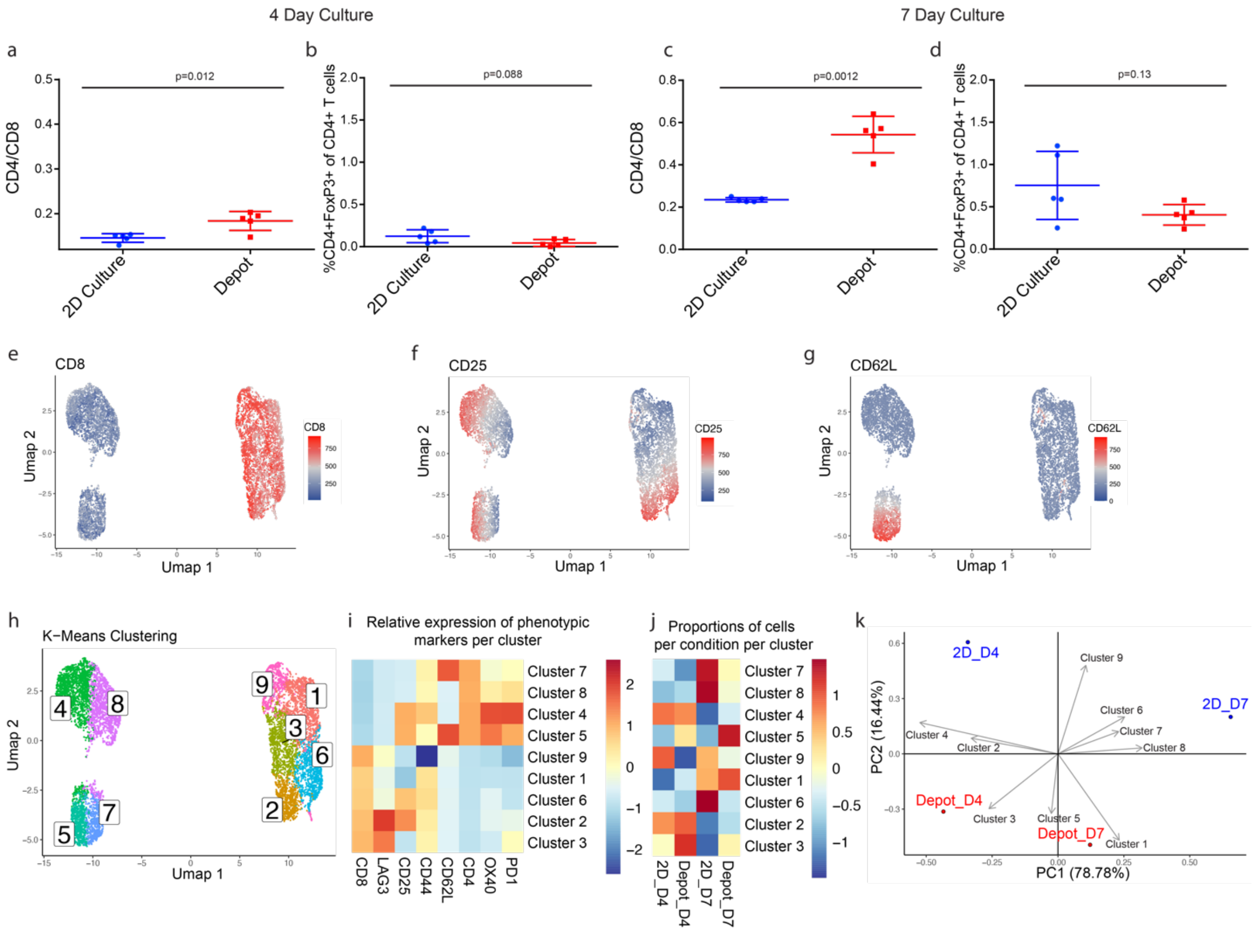

Supplementary Fig. 4. In vitro phenotyping of T cells in depots. T cells were first activated with dynabeads for 4 days, after which the dynabeads were removed. T cells were then cultured for additional 4 or 7 days in either depots or as 2D cultures. a. CD4/CD8 ratios of day 4 T cell cultures. b. Percentages of CD4+FOXP3+ present in day 4 T cell cultures. c-d. Same analysis as a-b for T cells harvested at day 7. P-values were determined by performing two-tailed unpaired t-test with Welch's correction. Data are mean ± s.d. from n=5 per condition e-k. Unsupervised phenotypic T cell analysis was performed for pooled T cells from days 4 and 7 harvest timepoints. e-g. Umap plots showing expression of the indicated markers. h. Umap plot overlaid with Kmeans clusters of T cells. i. Heatmap plot showing the average expression of the indicated T cell markers in each cluster after K-means analysis. j. Heatmap plot showing the proportion of cells in each condition represented in each cluster. k. PCA plot showing the relative similarities of the indicated conditions based on their T cell profiles. The PCA loadings show the effects of the individual clusters (from e) on the observed differences. The lengths of the arrows depict the magnitudes of the contributions while the directions show how the various clusters drive the locations of the conditions on the PCA plot. Data are pooled cells from n=5 per condition.

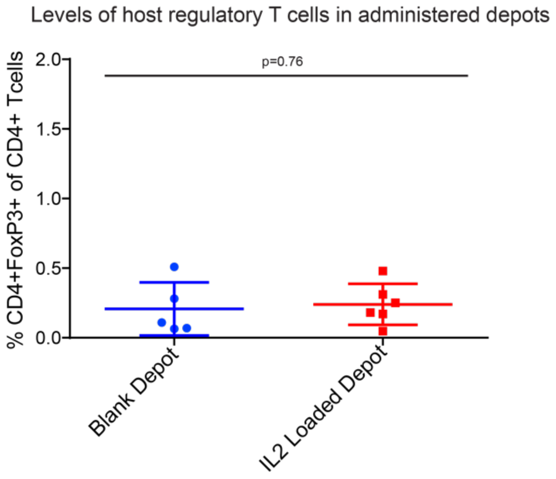

Supplementary Fig. 5. Host regulatory T cells in IL2 loaded depots in vivo. Plot showing the percentages of host CD4+FOXP3+ T cells in blank and IL2 loaded depots after 7 days of subcutaneous administration. P-value was determined by performing two-tailed unpaired t-test with Welch's correction. Data are mean ± s.d. from n=5 mice for blank depot and n=6 mice for IL2 loaded depot.

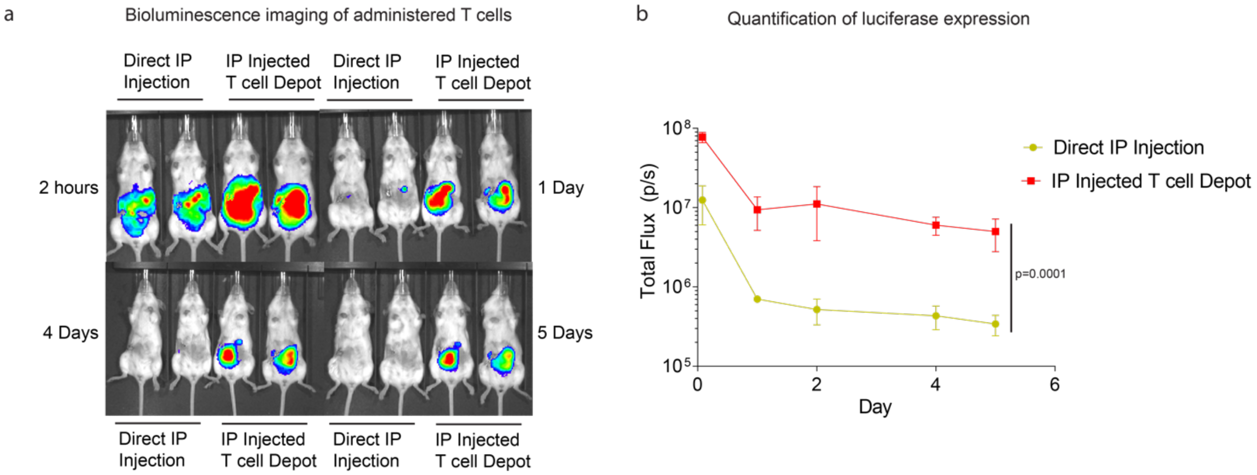

Supplementary Fig. 6. Analysis of local T cell persistence in the intraperitoneal space. T cells were isolated from spleens of luciferase expressing mice, activated in vitro, and either directly injected into the intraperitoneal (IP) space or loaded into depots before injection. a. Bioluminescence images of administered T cells over time from representative mice. b. Quantification of T cell luciferase expression over time. P-value was determined by two-way ANOVA with repeated measures. Data are mean ± s.d. from n=4.

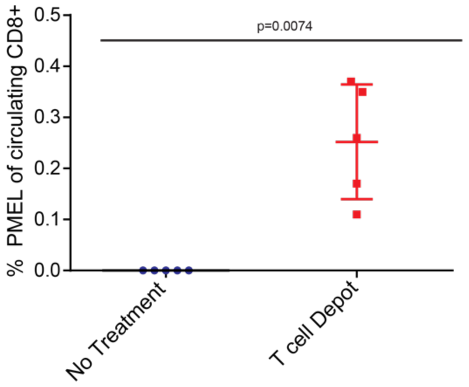

Supplementary Fig. 7. Detection of subcutaneously administered T cells in circulation. CD8+ T cells were isolated from spleens of pmel mice and activated with dynabeads for 4 days. T cells were then loaded into depots and subcutaneously injected into mice. Plot shows percentage of circulating pmel T cells 9 days after subcutaneous injection. P-value was determined by performing two-tailed unpaired t-test with Welch's correction. Data are mean ± s.d. from n=5 mice per condition.

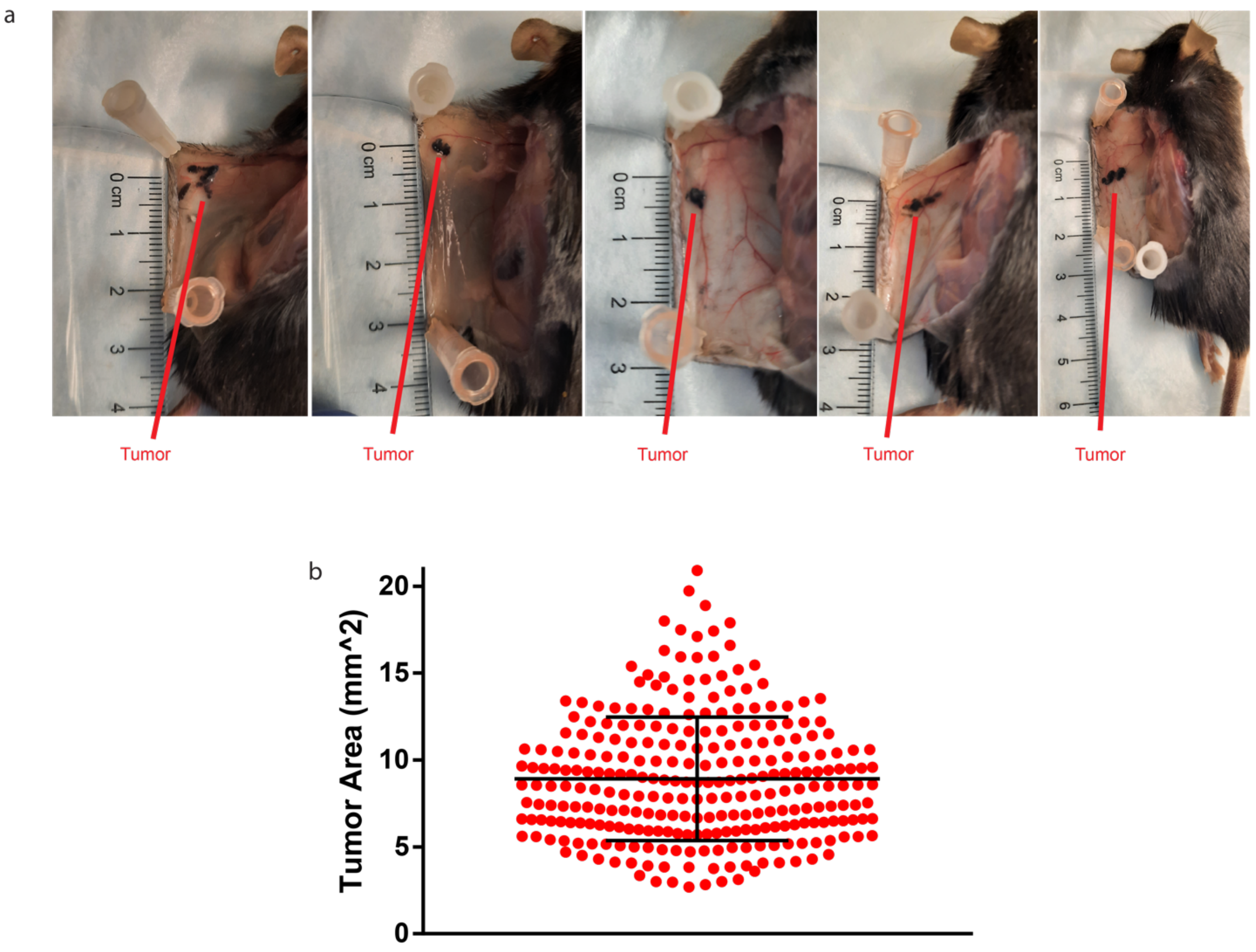

Supplementary Fig. 8. a. Representative images of established tumors before treatment. b. Measured tumor areas for 270 mice 24 hours before treatment. Data are mean ± s.d.

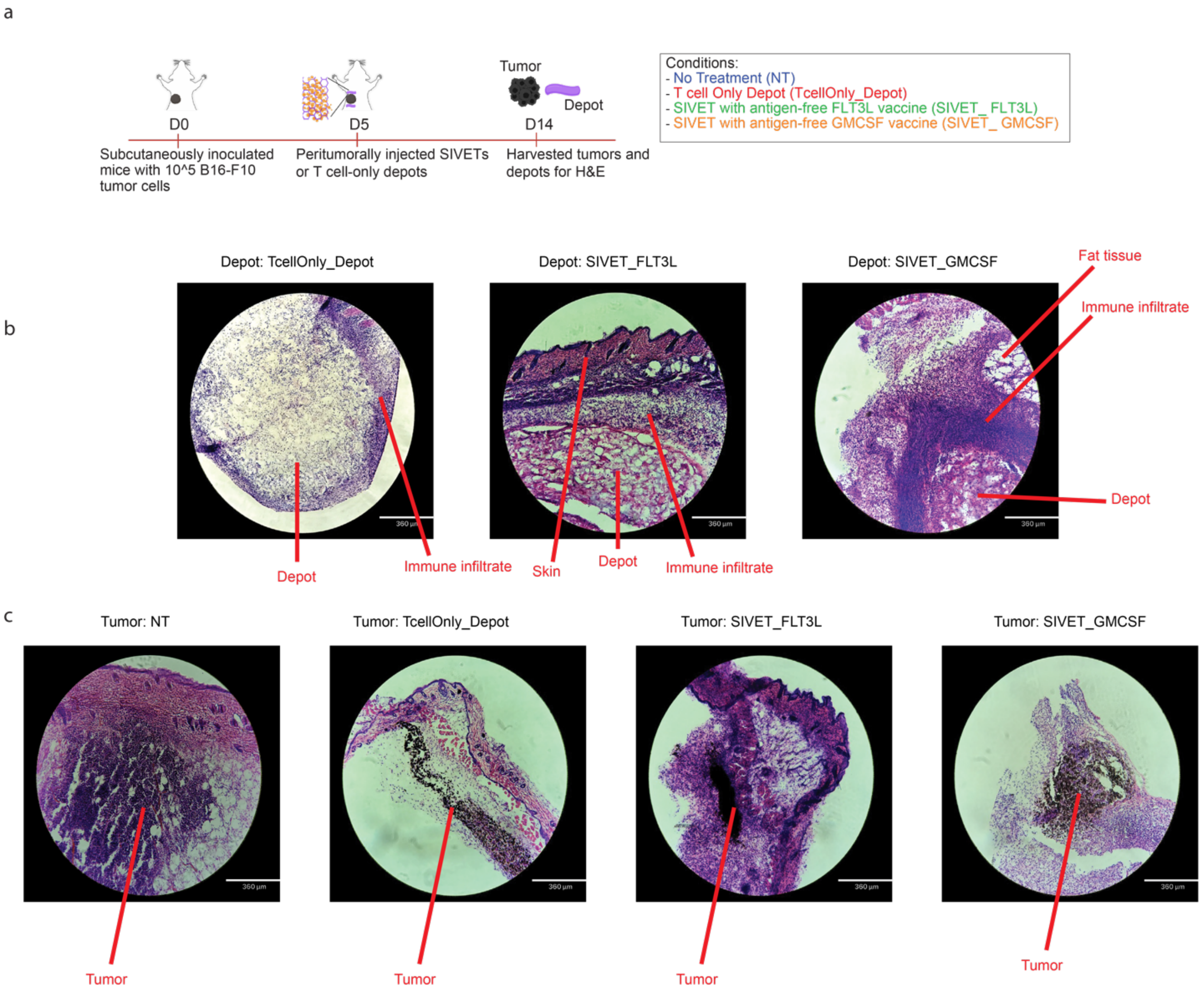

Supplementary Fig. 9 H&E images of depots and tumors after SIVET treatment. a. Schematic of experiment. Representative H&E images of depots (b) and tumors (c) for the indicated conditions. Regions of interest are labeled. Images are tiled acquisitions from a slide selected from 30 continuous sections each from 1 mouse per condition.

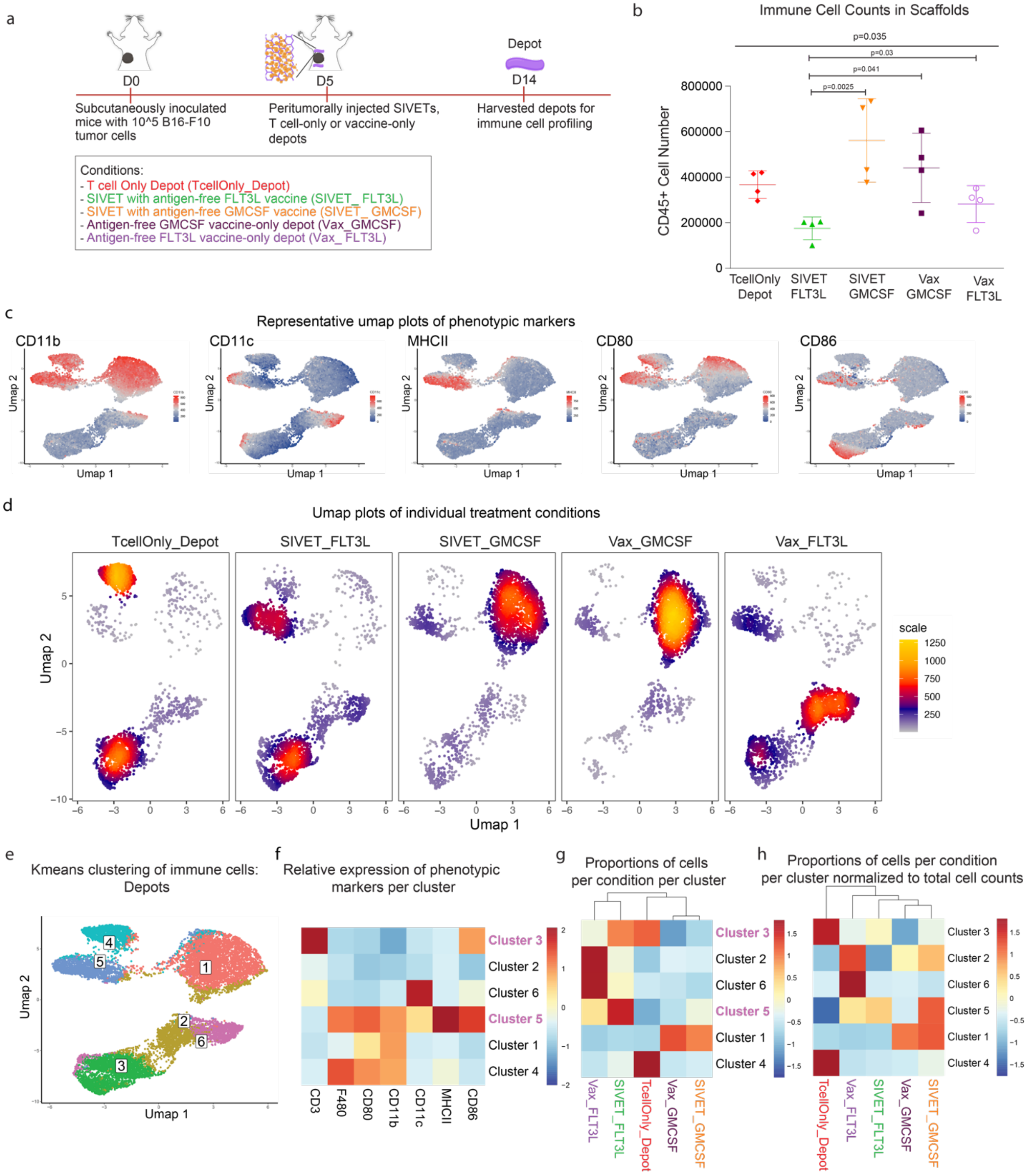

Supplementary Fig. 10 SIVETs enhance the relative levels of activated antigen-presenting cells in depots. a. Schematic of experiment. b. Total counts of immune cells that infiltrated depots per condition. P-values determined by two-tailed one-way ANOVA with Geisser-Greenhouse correction. Data are mean ± s.d. from n=4 mice per condition. c. Umap plots showing expression of the indicated markers. d. Umap density plots of individual treatment conditions showing distinct localization of cells based on treatment group. Denser (hot) regions indicate more cells. e. Umap plot overlaid with Kmeans clusters of immune cells in depots. f. Heatmap plot showing the average expression of the indicated immune cell markers in each cluster after K-means analysis. g. Heatmap plot showing the proportion of cells in each condition represented in each cluster. Some clusters of interest are highlighted. Data

are pooled cells from n=3 mice per condition. h. Heatmap plot showing the proportion of cells in each condition represented in each cluster, normalized to the total cell counts in (b). Data are pooled cells from n=3 mice per condition.

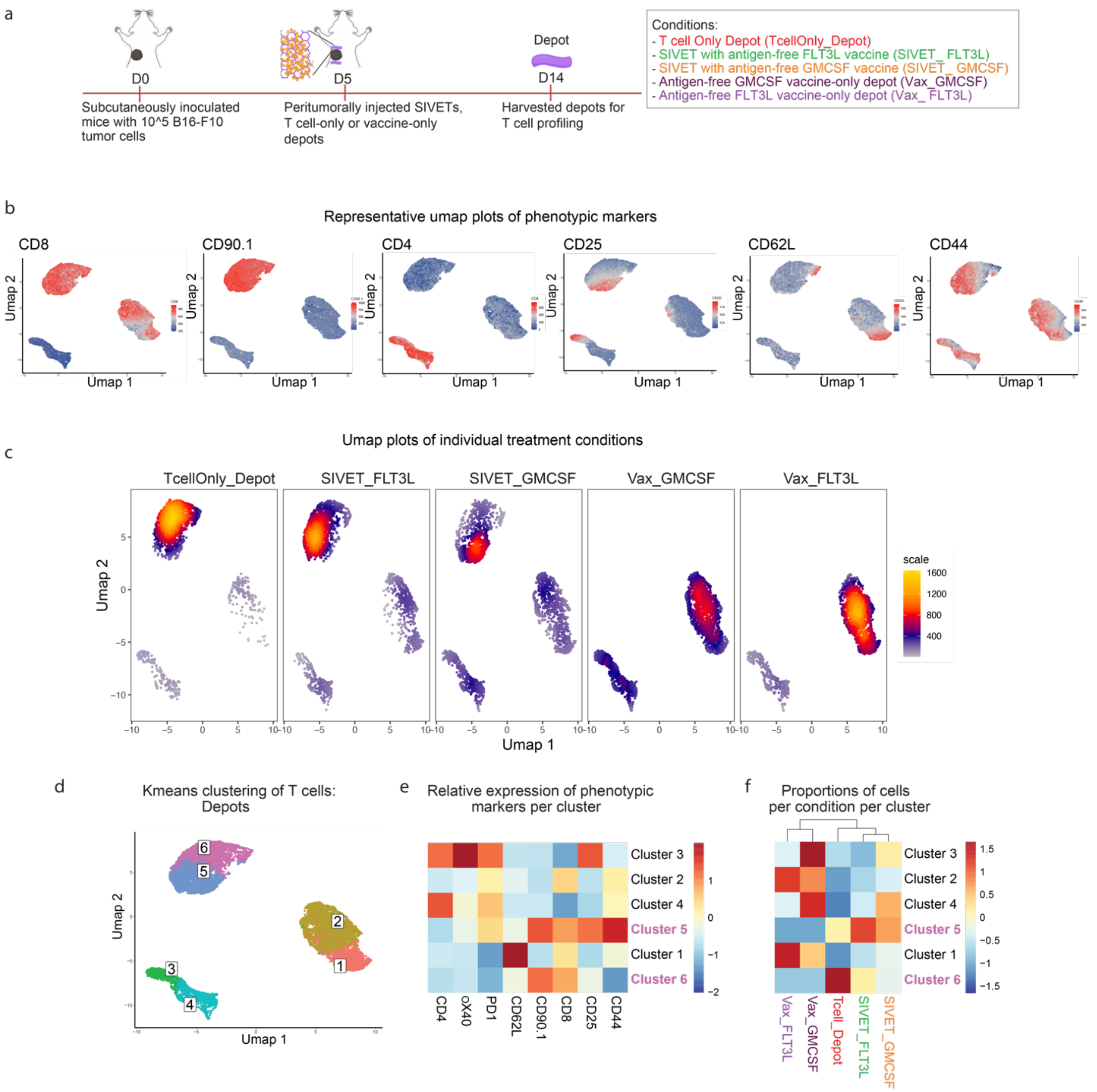

Supplementary Fig. 11 SIVETs prolong activation of adoptively transferred T cells in depots. a. Schematic of experiment. b. Umap plots showing expression of the indicated markers. c. Umap density plots of individual treatment conditions showing distinct localization of cells based on treatment group. Denser (hot) regions indicate more cells. d. Umap plot overlaid with Kmeans clusters of T cells in depots. e. Heatmap plot showing the average expression of the indicated T cell markers in each cluster after K-means analysis. f. Heatmap plot showing the proportion of cells in each condition represented in each cluster. Some clusters of interest are highlighted. Data are pooled cells from n=3 mice per condition.

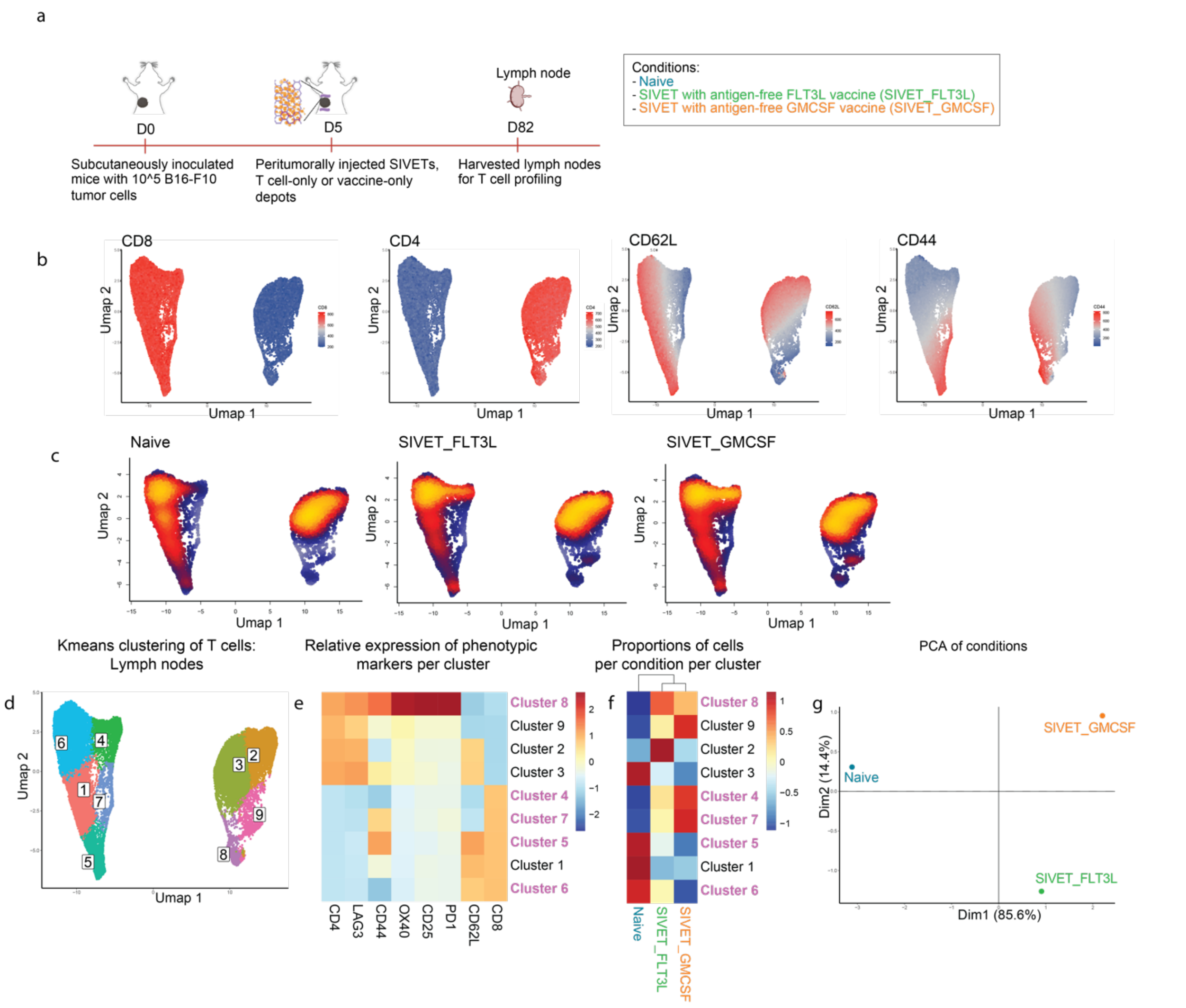

Supplementary Fig. 12 Characterization of long-term T cell profiles in lymph nodes after SIVET treatment. a. Schematic of experiment. b. Umap plots showing expression of the indicated T cell markers. c. 2D density plots showing umap of individual treatment conditions. Denser (hot) regions indicate more cells. d. Umap plot overlaid with Kmeans clusters of T cells in lymph nodes. e. Heatmap plot showing the average expression of the indicated T cell markers in each cluster after K-means analysis. f. Heatmap plot showing the proportion of T cells in each condition represented in each cluster. Some clusters of interest are highlighted. g. PCA plot showing relative similarities between the different treatment conditions. Data are pooled cells from n=3 mice per condition (SIVET_FLT3L and SIVET_GMCSF) and n=2 mice for naïve controls.

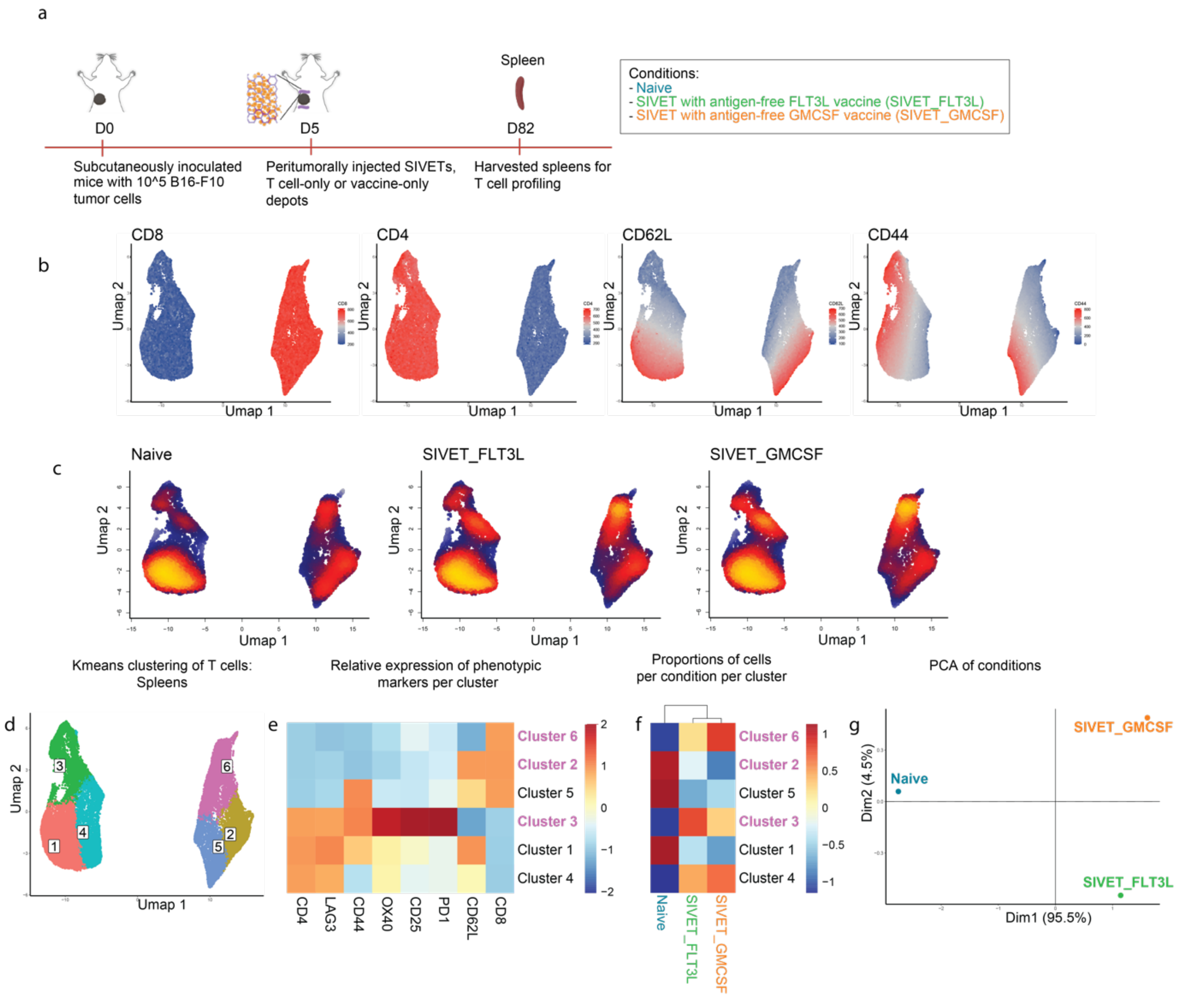

Supplementary Fig. 13 Characterization of long-term T cell profiles in spleens after SIVET treatment. a. Schematic of experiment. b. Umap plots showing expression of the indicated T cell markers. c. 2D density plots showing umap of individual treatment conditions. Denser (hot) regions indicate more cells. d. Umap plot overlaid with Kmeans clusters of T cells in spleens. e. Heatmap plot showing the average expression of the indicated T cell markers in each cluster after K-means analysis. f. Heatmap plot showing the proportion of T cells in each condition represented in each cluster. Some clusters of interest are highlighted. g. PCA plot showing relative similarities between the different treatment conditions. Data are pooled cells from n=3 mice per condition (SIVET_FLT3L and SIVET_GMCSF) and n=2 mice for naïve controls.

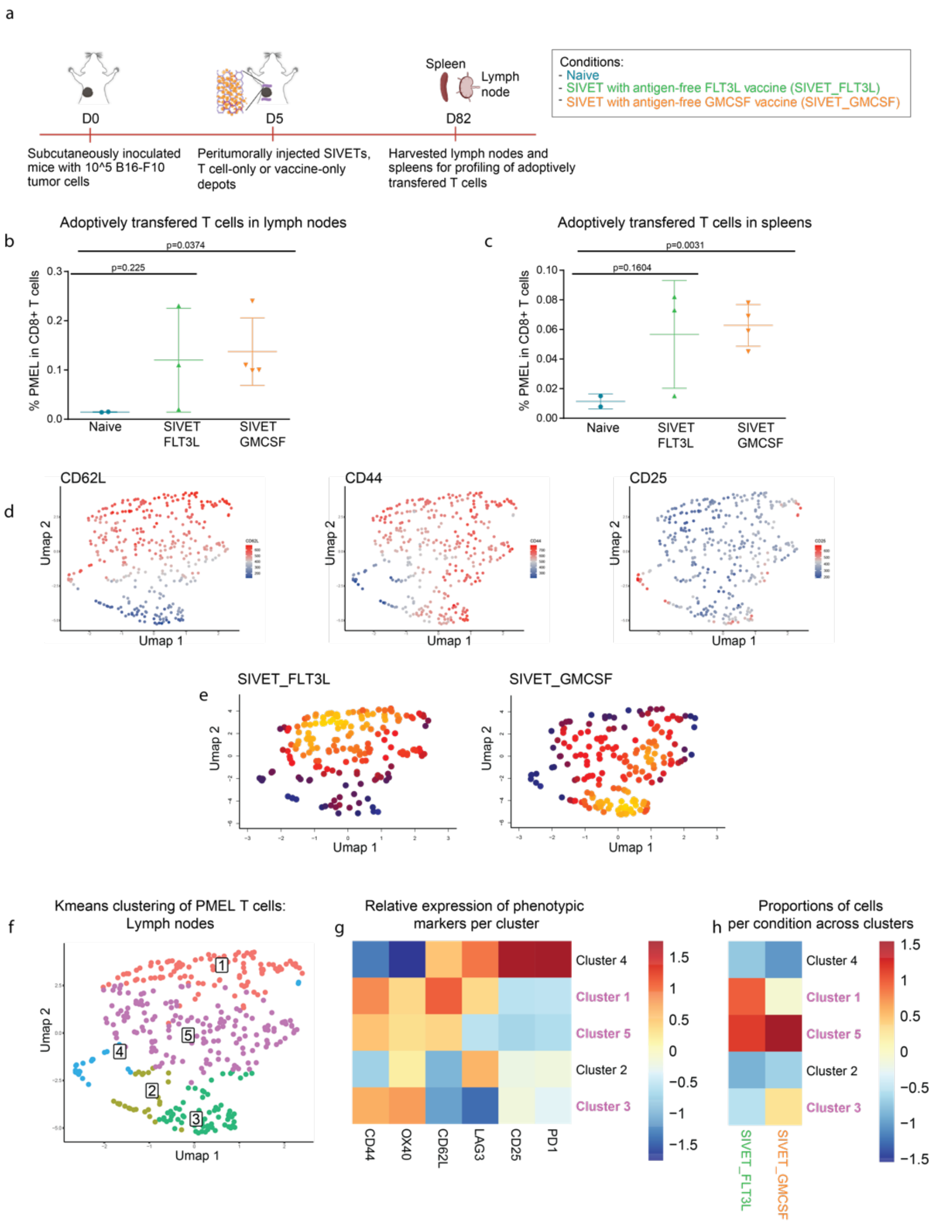

Supplementary Fig. 14 Characterization of long-term profiles of adoptively transferred T cells after SIVET treatment. a. Schematic of experiment. b-c. Proportions of adoptively transferred T cells in CD8+ populations in lymph nodes (b) and spleens (c) of long-term surviving mice. p-values determined by two-tailed unpaired t test with Welch's correction. Data are mean ± s.d. from n=3 mice per condition (SIVET_FLT3L and SIVET_GMCSF) and n=2 for naïve. d. Umap plots of adoptively transferred T cells from lymph nodes showing expression of the indicated T cell markers. e. 2D density plots showing umap of individual treatment conditions. Denser (hot) regions indicate more cells. f. Umap plot overlaid with Kmeans clusters of T cells. g. Heatmap plot showing the average expression of the indicated T cell markers in each cluster after K-means analysis. h. Heatmap plot showing the distribution of cells in each condition across the various clusters. Some clusters of interest are highlighted. Data are pooled cells from n=3 mice per condition.

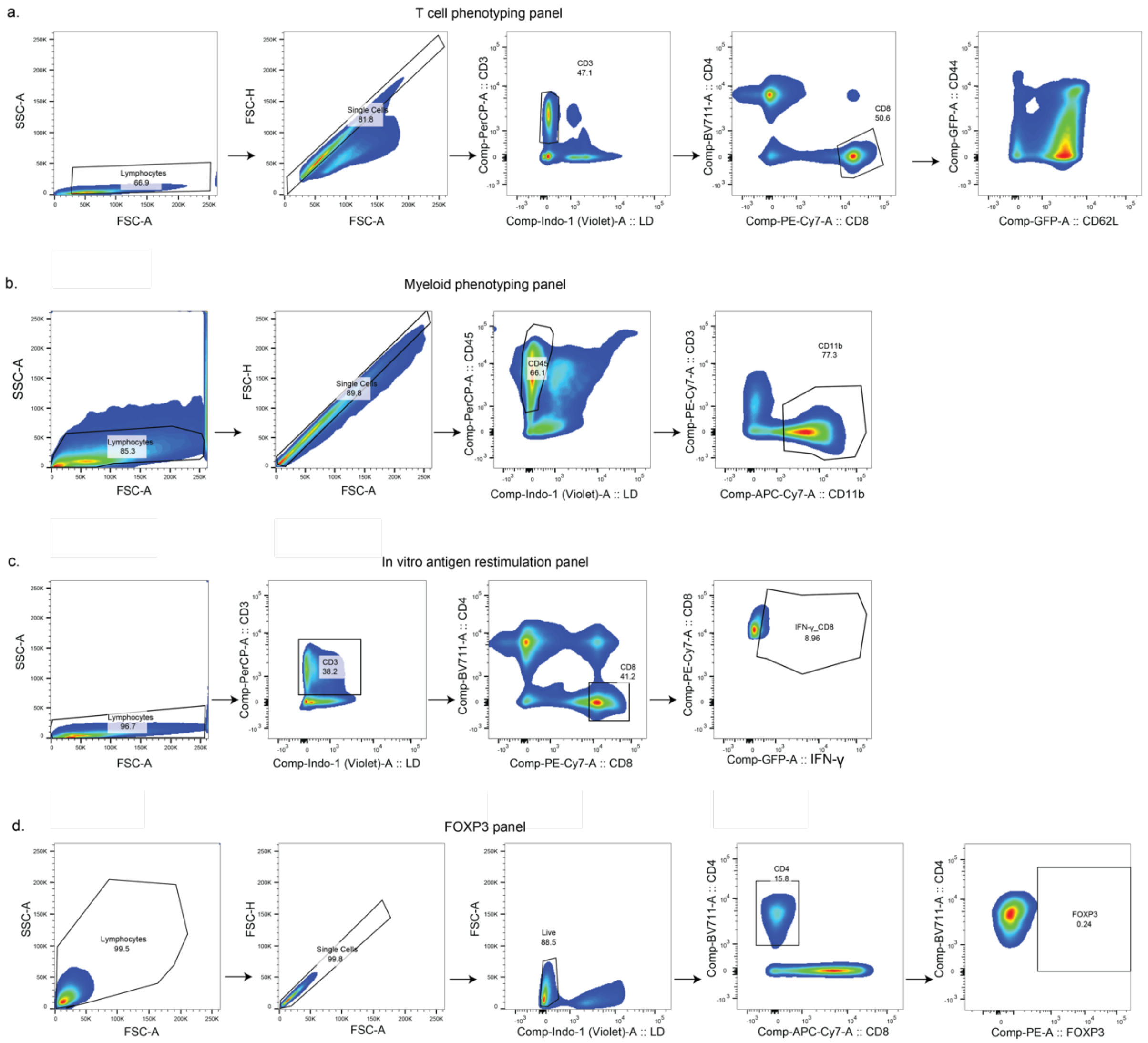

Supplementary Fig. 15 Flow cytometry gating strategies. Representative flow cytometry gating strategies for the various panels used for immune cell characterization.

Supplementary Table 1- Statistical Information for Main Figures

**Figure 1g: Two-way ANOVA with repeated measures**						
**CpG**						
Alpha	0.05					
Source of Variation	% of total variation	P value	P value summary	Significant?		
Interaction	13.38	< 0.0001	****	Yes		
Time	84.4	< 0.0001	****	Yes		
Column Factor	1.654	0.0007	***	Yes		
Subjects (matching)	0.07297	0.3313	ns	No		
ANOVA table	SS	DF	MS	F (DFn	DFd)	P value
Interaction	414.4	8	51.8	F (8	32) = 109.7	P < 0.0001
Time	2613	8	326.6	F (8	32) = 692.1	P < 0.0001
Column Factor	51.21	1	51.21	F (1	4) = 90.67	P = 0.0007
Subjects (matching)	2.259	4	0.5648	F (4	32) = 1.197	P = 0.3313
Residual	15.1	32	0.472			
Number of missing values	0					
**IL2**						
Alpha	0.05					
Source of Variation	% of total variation	P value	P value summary	Significant?		
Interaction	57.14	< 0.0001	****	Yes		
Time	39.92	< 0.0001	****	Yes		
Column Factor	1.48	0.0084	**	Yes		
Subjects (matching)	0.2527	0.3145	ns	No		
ANOVA table	SS	DF	MS	F (DFn	DFd)	P value
Interaction	7.48E+06	6	1.25E+06	F (6	24) = 189.3	P < 0.0001
Time	5.23E+06	6	871497	F (6	24) = 132.3	P < 0.0001
Column Factor	193883	1	193883	F (1	4) = 23.43	P = 0.0084
Subjects (matching)	33101	4	8275	F (4	24) = 1.256	P = 0.3145
Residual	158141	24	6589			
Number of missing values	0					
**GMCSF**						
Alpha	0.05					
Source of Variation	% of total variation	P value	P value summary	Significant?		
Interaction	45.34	< 0.0001	****	Yes		
Time	45.99	< 0.0001	****	Yes		
Column Factor	7.608	0.0083	**	Yes		
Subjects (matching)	0.1279	0.4663	ns	No		
ANOVA table	SS	DF	MS	F (DFn	DFd)	P value
Interaction	2.33E+06	6	388131	F (6	12) = 96.13	P < 0.0001
Time	2.36E+06	6	393696	F (6	12) = 97.50	P < 0.0001
Column Factor	390833	1	390833	F (1	2) = 119.0	P = 0.0083
Subjects (matching)	6571	2	3285	F (2	12) = 0.8137	P = 0.4663
Residual	48453	12	4038			
Number of missing values	0					
**Figure 1j: Two-way ANOVA with repeated measures**						
Alpha	0.05					
Source of Variation	% of total variation	P value	P value summary	Significant?		
Interaction	5.801	0.0255	*	Yes		
Time	62.25	<0.0001	****	Yes		
Column Factor	19.02	0.0018	**	Yes		
Subjects (matching)	4.077	0.2748	ns	No		
ANOVA table	SS	DF	MS	F (DFn	DFd)	P value
Interaction	9.27E+13	3	3.09E+13	F (3	18) = 3.932	P = 0.0255
Time	9.94E+14	3	3.31E+14	F (3	18) = 42.20	P < 0.0001
Column Factor	3.04E+14	1	3.04E+14	F (1	6) = 27.99	P = 0.0018
Subjects (matching)	6.51E+13	6	1.09E+13	F (6	18) = 1.382	P = 0.2748
Residual	1.41E+14	18	7.85E+12			
Number of missing values	0					
**Figure 2c :Log-Rank (Mantel-Cox) Test**						
Comparison of Survival Curves	**No Treament vs Empty Cryogel**					
Log-rank (Mantel-Cox) test						
Chi square	0.0557					
df	1					
P value	0.8134					
P value summary	ns					
Are the survival curves sig different?	No					
Gehan-Breslow-Wilcoxon test						
Chi square	0.02928					
df	1					
P value	0.8641					
P value summary	ns					
Are the survival curves sig different?	No					
Median survival						
Tumor Only Control	26					
Empty Cryogel	25					
Ratio (and its reciprocal)	1.04	0.9615				
95% CI of ratio	0.5201 to 2.080	0.4809 to 1.923				

Hazard Ratio (Mantel-Haenszel)		
Ratio (and its reciprocal)	0.9056	1.104
95% CI of ratio	0.3976 to 2.063	0.4848 to 2.515
Hazard Ratio (logrank)		
Ratio (and its reciprocal)	0.9321	1.073
95% CI of ratio	0.4527 to 1.812	0.5520 to 2.209
Comparison of Survival Curves	**IV Injected T cells vs T cell Depot**	
Log-rank (Mantel-Cox) test		
Chi square	23.52	
df	1	
P value	< 0.0001	
P value summary	****	
Are the survival curves sig different?	Yes	
Gehan-Breslow-Wilcoxon test		
Chi square	17.73	
df	1	
P value	< 0.0001	
P value summary	****	
Are the survival curves sig different?	Yes	
Median survival		
IV Injected T cells with IL2	39	
T cells In Cryogel	58	
Ratio (and its reciprocal)	0.6724	1.487
95% CI of ratio	0.2878 to 1.571	0.6365 to 3.475
Hazard Ratio (Mantel-Haenszel)		
Ratio (and its reciprocal)	12.33	0.08109
95% CI of ratio	4.468 to 34.04	0.02938 to 0.2238
Hazard Ratio (logrank)		
Ratio (and its reciprocal)	5.231	0.1912
95% CI of ratio	5.042 to 30.17	0.03315 to 0.1983
Comparison of Survival Curves	**Direct Peritumoral Injection vs T cell Depot**	
Log-rank (Mantel-Cox) test		
Chi square	9.155	
df	1	
P value	0.0025	
P value summary	**	
Are the survival curves sig different?	Yes	
Gehan-Breslow-Wilcoxon test		
Chi square	10.83	
df	1	
P value	0.001	
P value summary	***	
Are the survival curves sig different?	Yes	
Median survival		
Direct Peritumoral Injection w	41.5	
T cells In Cryogel	58	
Ratio (and its reciprocal)	0.7155	1.398
95% CI of ratio	0.3034 to 1.688	0.5925 to 3.296
Hazard Ratio (Mantel-Haenszel)		
Ratio (and its reciprocal)	4.076	0.2453
95% CI of ratio	1.640 to 10.13	0.09872 to 0.6096
Hazard Ratio (logrank)		
Ratio (and its reciprocal)	3.207	0.3118
95% CI of ratio	1.747 to 9.508	0.1052 to 0.5722
Comparison of Survival Curves	**No Treatment vs T cell Depot**	
Log-rank (Mantel-Cox) test		
Chi square	33.07	
df	1	
P value	< 0.0001	
P value summary	****	
Are the survival curves sig different?	Yes	
Gehan-Breslow-Wilcoxon test		
Chi square	27.94	
df	1	
P value	< 0.0001	
P value summary	****	
Are the survival curves sig different?	Yes	
Median survival		
Tumor Only Control	26	
T cells In Cryogel	58	
Ratio (and its reciprocal)	0.4483	2.231
95% CI of ratio	0.1918 to 1.047	0.9547 to 5.212
Hazard Ratio (Mantel-Haenszel)		
Ratio (and its reciprocal)	24.4	0.04098
95% CI of ratio	8.214 to 72.49	0.01379 to 0.1217
Hazard Ratio (logrank)		
Ratio (and its reciprocal)	6.499	0.1539
95% CI of ratio	9.502 to 62.67	0.01596 to 0.1052
**Figure 2d :Log-Rank (Mantel-Cox) Test**		
Comparison of Survival Curves	**No Treatment vs T cell Depot**	
Log-rank (Mantel-Cox) test		

Chi square	9.106					
df	1					
P value	0.0025					
P value summary	**					
Are the survival curves sig different?	Yes					
Gehan-Breslow-Wilcoxon test						
Chi square	7.717					
df	1					
P value	0.0055					
P value summary	**					
Are the survival curves sig different?	Yes					
Median survival						
Tumor Only Control	24					
T cells In Cryogel	38					
Ratio (and its reciprocal)	0.6316	1.583				
95% CI of ratio	0.2437 to 1.637	0.6109 to 4.104				
Hazard Ratio (Mantel-Haenszel)						
Ratio (and its reciprocal)	6.769	0.1477				
95% CI of ratio	1.955 to 23.44	0.04266 to 0.5116				
Hazard Ratio (logrank)						
Ratio (and its reciprocal)	3.387	0.2952				
95% CI of ratio	2.256 to 20.31	0.04923 to 0.4433				
Comparison of Survival Curves	**Contralateral injection_T cell depot vs T cell depot**					
Log-rank (Mantel-Cox) test						
Chi square	6.328					
df	1					
P value	0.0119					
P value summary	*					
Are the survival curves sig different?	Yes					
Gehan-Breslow-Wilcoxon test						
Chi square	4.195					
df	1					
P value	0.0405					
P value summary	*					
Are the survival curves sig different?	Yes					
Median survival						
T cells In Cryogel_Contralater	26					
T cells In Cryogel	38					
Ratio (and its reciprocal)	0.6842	1.462				
95% CI of ratio	0.2640 to 1.773	0.5639 to 3.788				
Hazard Ratio (Mantel-Haenszel)						
Ratio (and its reciprocal)	4.897	0.2042				
95% CI of ratio	1.420 to 16.89	0.05922 to 0.7041				
Hazard Ratio (logrank)						
Ratio (and its reciprocal)	2.687	0.3722				
95% CI of ratio	1.727 to 13.89	0.07201 to 0.5790				
Comparison of Survival Curves	**IV Injected T cells vs T cell depot**					
Log-rank (Mantel-Cox) test						
Chi square	4.512					
df	1					
P value	0.0337					
P value summary	*					
Are the survival curves sig different?	Yes					
Gehan-Breslow-Wilcoxon test						
Chi square	2.902					
df	1					
P value	0.0885					
P value summary	ns					
Are the survival curves sig different?	No					
Median survival						
IV Injected T cells with IL2	29					
T cells In Cryogel	38					
Ratio (and its reciprocal)	0.7632	1.31				
95% CI of ratio	0.2944 to 1.978	0.5056 to 3.396				
Hazard Ratio (Mantel-Haenszel)						
Ratio (and its reciprocal)	3.442	0.2905				
95% CI of ratio	1.100 to 10.77	0.09288 to 0.9089				
Hazard Ratio (logrank)						
Ratio (and its reciprocal)	2.45	0.4082				
95% CI of ratio	1.237 to 9.580	0.1044 to 0.8087				
Comparison of Survival Curves	**Direct Peritumoral Injection vs T cell depot**					
Log-rank (Mantel-Cox) test						
Chi square	3.849					
df	1					
P value	0.0498					
P value summary	*					
Are the survival curves sig different?	Yes					
Gehan-Breslow-Wilcoxon test						
Chi square	2.723					
df	1					
P value	0.0989					
P value summary	ns					
Are the survival curves sig different?	No					

Median survival						
Direct Peritumoral Injection w	28					
T cells In Cryogel	38					
Ratio (and its reciprocal)	0.7368	1.357				
95% CI of ratio	0.2843 to 1.910	0.5236 to 3.518				
Hazard Ratio (Mantel-Haenszel)						
Ratio (and its reciprocal)	3.025	0.3306				
95% CI of ratio	1.001 to 9.138	0.1094 to 0.9989				
Hazard Ratio (logrank)						
Ratio (and its reciprocal)	2.338	0.4276				
95% CI of ratio	1.096 to 8.346	0.1198 to 0.9124				
**Figure 3b :Two-tailed one-way ANOVA with Geisser-Greenhouse correction**						
ANOVA summary						
F	8.867					
P value	0.0002					
P value summary	***					
Are differences among means statistically significant? (P < 0.05)	Yes					
R square	0.7112					
Brown-Forsythe test						
F (DFn	DFd)	1.890 (5	18)			
P value	0.1462					
P value summary	ns					
Significantly different standard deviations? (P < 0.05)	No					
Bartlett's test						
Bartlett's statistic (corrected)	11.73					
P value	0.0387					
P value summary	*					
Significantly different standard deviations? (P < 0.05)	Yes					
ANOVA table	SS	DF	MS	F (DFn	DFd)	P value
Treatment (between columns)	43.15	5	8.629	F (5	18) = 8.867	P = 0.0002
Residual (within columns)	17.52	18	0.9732			
Total	60.66	23				
Data summary						
Number of treatments (columns)	6					
Number of values (total)	24					
**Figure 3c :Two-tailed one-way ANOVA with Geisser-Greenhouse correction**						
ANOVA summary						
F	3.307					
P value	0.0272					
P value summary	*					
Are differences among means statistically significant? (P < 0.05)	Yes					
R square	0.4788					
Brown-Forsythe test						
F (DFn	DFd)	0.6784 (5	18)			
P value	0.6454					
P value summary	ns					
Significantly different standard deviations? (P < 0.05)	No					
Bartlett's test						
Bartlett's statistic (corrected)	9.358					
P value	0.0956					
P value summary	ns					
Significantly different standard deviations? (P < 0.05)	No					
ANOVA table	SS	DF	MS	F (DFn	DFd)	P value
Treatment (between columns)	1.905	5	0.3809	F (5	18) = 3.307	P = 0.0272
Residual (within columns)	2.073	18	0.1152			
Total	3.978	23				
Data summary						
Number of treatments (columns)	6					
Number of values (total)	24					
**Figure 3d :Two-tailed one-way ANOVA with Geisser-Greenhouse correction**						
ANOVA summary						
F	10.73					
P value	< 0.0001					
P value summary	****					
Are differences among means statistically significant? (P < 0.05)	Yes					
R square	0.7488					
Brown-Forsythe test						
F (DFn	DFd)	1.850 (5	18)			
P value	0.1537					
P value summary	ns					
Significantly different standard deviations? (P < 0.05)	No					
Bartlett's test						
Bartlett's statistic (corrected)	6.649					
P value	0.2481					
P value summary	ns					
Significantly different standard deviations? (P < 0.05)	No					
ANOVA table	SS	DF	MS	F (DFn	DFd)	P value
Treatment (between columns)	29.93	5	5.987	F (5	18) = 10.73	P < 0.0001
Residual (within columns)	10.04	18	0.558			
Total	39.98	23				
Data summary						

Number of treatments (columns)	6					
Number of values (total)	24					
**Figure 3e :Two-tailed one-way ANOVA with Geisser-Greenhouse correction**						
ANOVA summary						
F	2.464					
P value	0.0722					
P value summary	ns					
Are differences among means statistically significant? (P < 0.05)	No					
R square	0.4063					
Brown-Forsythe test						
F (DFn	DFd)	0.6667 (5	18)			
P value	0.6535					
P value summary	ns					
Significantly different standard deviations? (P < 0.05)	No					
Bartlett's test						
Bartlett's statistic (corrected)	9.336					
P value	0.0964					
P value summary	ns					
Significantly different standard deviations? (P < 0.05)	No					
ANOVA table	SS	DF	MS	F (DFn	DFd)	P value
Treatment (between columns)	0.8592	5	0.1718	F (5	18) = 2.464	P = 0.0722
Residual (within columns)	1.256	18	0.06975			
Total	2.115	23				
Data summary						
Number of treatments (columns)	6					
Number of values (total)	24					
**Figure 4b :Two-tailed one-way ANOVA with Geisser-Greenhouse correction**						
ANOVA summary						
F	3.722					
P value	0.0289					
P value summary	*					
Are differences among means statistically significant? (P < 0.05)	Yes					
R square	0.608					
Brown-Forsythe test						
F (DFn, DFd)	0.7112 (5, 12)					
P value	0.6266					
P value summary	ns					
Significantly different standard deviations? (P < 0.05)	No					
Bartlett's test						
Bartlett's statistic (corrected)						
P value						
P value summary						
Significantly different standard deviations? (P < 0.05)						
ANOVA table	SS	DF	MS	F (DFn, DFd)	P value	
Treatment (between columns)	1246000000	5	249300000	F (5, 12) = 3.7	P = 0.0289	
Residual (within columns)	803700000	12	66980000			
Total	2050000000	17				
Data summary						
Number of treatments (columns)	6					
Number of values (total)	18					
**Figure 5b :Two-tailed one-way ANOVA with Geisser-Greenhouse correction**						
ANOVA summary						
F	2.834					
P value	0.0646					
P value summary	ns					
Are differences among means statistically significant? (P < 0.05)	No					
R square	0.5415					
Brown-Forsythe test						
F (DFn, DFd)	0.6627 (5, 12)					
P value	0.6587					
P value summary	ns					
Significantly different standard deviations? (P < 0.05)	No					
Bartlett's test						
Bartlett's statistic (corrected)						
P value						
P value summary						
Significantly different standard deviations? (P < 0.05)						
ANOVA table	SS	DF	MS	F (DFn, DFd)	P value	
Treatment (between columns)	36840000	5	7368000	F (5, 12) = 2.8	P = 0.0646	
Residual (within columns)	31200000	12	2600000			
Total	68040000	17				
Data summary						
Number of treatments (columns)	6					
Number of values (total)	18					
**Figure 6c :Log-rank (Mantel-Cox) test**						
Comparison of Survival Curves	**NT vs T cell depot**					
Log-rank (Mantel-Cox) test						
Chi square	7.942					
df	1					
P value	0.0048					
P value summary	**					
Are the survival curves sig different?	Yes					

Gehan-Breslow-Wilcoxon test						
Chi square	6.18					
df	1					
P value	0.0129					
P value summary	*					
Are the survival curves sig different?	Yes					
Median survival						
Tumor Only Control	22					
T cells in Cryogel with IL2	33					
Ratio (and its reciprocal)	0.6667	1.5				
95% CI of ratio	0.2587 to 1.718	0.5820 to 3.866				
Hazard Ratio (Mantel-Haenszel)						
Ratio (and its reciprocal)	4.447	0.2249				
95% CI of ratio	1.575 to 12.55	0.07966 to 0.6348				
Hazard Ratio (logrank)						
Ratio (and its reciprocal)	2.925	0.3419				
95% CI of ratio	1.885 to 10.49	0.09535 to 0.5304				
Comparison of Survival Curves	**NT vs SIVETs**					
Log-rank (Mantel-Cox) test						
Chi square	56.06					
df	1					
P value	< 0.0001					
P value summary	****					
Are the survival curves sig different?	Yes					
Gehan-Breslow-Wilcoxon test						
Chi square	51.01					
df	1					
P value	< 0.0001					
P value summary	****					
Are the survival curves sig different?	Yes					
Median survival						
Tumor Only Control	22					
SIVET	Undefined					
Hazard Ratio (Mantel-Haenszel)						
Ratio (and its reciprocal)	123.3	0.008111				
95% CI of ratio	34.96 to 434.8	0.002300 to 0.02860				
Hazard Ratio (logrank)						
Ratio (and its reciprocal)	10.25	0.09756				
95% CI of ratio	38.90 to 390.7	0.002559 to 0.02571				
Comparison of Survival Curves	**NT vs Vax only**					
Log-rank (Mantel-Cox) test						
Chi square	4.954					
df	1					
P value	0.026					
P value summary	*					
Are the survival curves sig different?	Yes					
Gehan-Breslow-Wilcoxon test						
Chi square	3.609					
df	1					
P value	0.0575					
P value summary	ns					
Are the survival curves sig different?	No					
Median survival						
Tumor Only Control	22					
Vaccine	25.5					
Ratio (and its reciprocal)	0.8627	1.159				
95% CI of ratio	0.4218 to 1.765	0.5666 to 2.371				
Hazard Ratio (Mantel-Haenszel)						
Ratio (and its reciprocal)	2.709	0.3691				
95% CI of ratio	1.126 to 6.516	0.1535 to 0.8878				
Hazard Ratio (logrank)						
Ratio (and its reciprocal)	1.991	0.5022				
95% CI of ratio	1.269 to 5.784	0.1729 to 0.7882				
Comparison of Survival Curves	**T cell depot vs SIVETs**					
Log-rank (Mantel-Cox) test						
Chi square	12.06					
df	1					
P value	0.0005					
P value summary	***					
Are the survival curves sig different?	Yes					
Gehan-Breslow-Wilcoxon test						
Chi square	11.82					
df	1					
P value	0.0006					
P value summary	***					
Are the survival curves sig different?	Yes					
Median survival						
T cells in Cryogel with IL2	33					
SIVET	Undefined					
Hazard Ratio (Mantel-Haenszel)						
Ratio (and its reciprocal)	16.97	0.05894				
95% CI of ratio	3.431 to 83.88	0.01192 to 0.2914				

Hazard Ratio (logrank)						
Ratio (and its reciprocal)	5.074	0.1971				
95% CI of ratio	3.496 to 82.33	0.01215 to 0.2860				
Comparison of Survival Curves	**T cell depot vs Vax only**					
Log-rank (Mantel-Cox) test						
Chi square	1.756					
df	1					
P value	0.1851					
P value summary	ns					
Are the survival curves sig different?	No					
Gehan-Breslow-Wilcoxon test						
Chi square	1.851					
df	1					
P value	0.1737					
P value summary	ns					
Are the survival curves sig different?	No					
Median survival						
T cells in Cryogel with IL2	33					
Vaccine	25.5					
Ratio (and its reciprocal)	1.294	0.7727				
95% CI of ratio	0.5021 to 3.335	0.2998 to 1.992				
Hazard Ratio (Mantel-Haenszel)						
Ratio (and its reciprocal)	0.531	1.883				
95% CI of ratio	0.2083 to 1.354	0.7385 to 4.802				
Hazard Ratio (logrank)						
Ratio (and its reciprocal)	0.5572	1.795				
95% CI of ratio	0.2231 to 1.264	0.7911 to 4.482				
Comparison of Survival Curves	**SIVET vs Vax only**					
Log-rank (Mantel-Cox) test						
Chi square	36.01					
df	1					
P value	< 0.0001					
P value summary	****					
Are the survival curves sig different?	Yes					
Gehan-Breslow-Wilcoxon test						
Chi square	34.76					
df	1					
P value	< 0.0001					
P value summary	****					
Are the survival curves sig different?	Yes					
Median survival						
SIVET	Undefined					
Vaccine	25.5					
Hazard Ratio (Mantel-Haenszel)						
Ratio (and its reciprocal)	0.03472	28.8				
95% CI of ratio	0.01159 to 0.1040	9.611 to 86.32				
Hazard Ratio (logrank)						
Ratio (and its reciprocal)	0.1303	7.673				
95% CI of ratio	0.01221 to 0.09871	10.13 to 81.89				
Comparison of Survival Curves	**SIVET FLT3L vs SIVET GMCSF**					
Log-rank (Mantel-Cox) test						
Chi square	1.042					
df	1					
P value	0.3075					
P value summary	ns					
Are the survival curves sig different?	No					
Gehan-Breslow-Wilcoxon test						
Chi square	0.7299					
df	1					
P value	0.3929					
P value summary	ns					
Are the survival curves sig different?	No					
Median survival						
T cells in Cryogel with IL2_ A	Undefined					
T cells in Cryogel with IL2_ A	Undefined					
Hazard Ratio (Mantel-Haenszel)						
Ratio (and its reciprocal)	1.981	0.5048				
95% CI of ratio	0.5330 to 7.363	0.1358 to 1.876				
Hazard Ratio (logrank)						
Ratio (and its reciprocal)	2.021	0.4948				
95% CI of ratio	0.5363 to 7.318	0.1367 to 1.865				
**Figure 6e :Log-rank (Mantel-Cox) test**						
Comparison of Survival Curves	**NT vs SIVET FLT3L**					
Log-rank (Mantel-Cox) test						
Chi square	7.849					
df	1					
P value	0.0051					
P value summary	**					
Are the survival curves sig different?	Yes					
Gehan-Breslow-Wilcoxon test						
Chi square	7.427					

df	1	
P value	0.0064	
P value summary	**	
Are the survival curves sig different?	Yes	
Median survival		
No Treatment Control	21	
T cell Delivery_FLT3L Vaccine	27	
Ratio (and its reciprocal)	0.7778	1.286
95% CI of ratio	0.2961 to 2.043	0.4894 to 3.378
Hazard Ratio (Mantel-Haenszel)		
Ratio (and its reciprocal)	5.572	0.1795
95% CI of ratio	1.675 to 18.53	0.05395 to 0.5969
Hazard Ratio (logrank)		
Ratio (and its reciprocal)	3.043	0.3286
95% CI of ratio	2.010 to 15.45	0.06474 to 0.4974
Comparison of Survival Curves	**NT vs SIVET GMCSF**	
Log-rank (Mantel-Cox) test		
Chi square	20.86	
df	1	
P value	< 0.0001	
P value summary	****	
Are the survival curves sig different?	Yes	
Gehan-Breslow-Wilcoxon test		
Chi square	19.11	
df	1	
P value	< 0.0001	
P value summary	****	
Are the survival curves sig different?	Yes	
Median survival		
No Treatment Control	21	
T cell Delivery_GMCSF Vaccine	40	
Ratio (and its reciprocal)	0.525	1.905
95% CI of ratio	0.2133 to 1.292	0.7740 to 4.688
Hazard Ratio (Mantel-Haenszel)		
Ratio (and its reciprocal)	24.5	0.04082
95% CI of ratio	6.209 to 96.64	0.01035 to 0.1611
Hazard Ratio (logrank)		
Ratio (and its reciprocal)	4.96	0.2016
95% CI of ratio	7.657 to 78.36	0.01276 to 0.1306
Comparison of Survival Curves	**SIVET FLT3L vs SIVET GMCSF**	
Log-rank (Mantel-Cox) test		
Chi square	0.7751	
df	1	
P value	0.3787	
P value summary	ns	
Are the survival curves sig different?	No	
Gehan-Breslow-Wilcoxon test		
Chi square	2.04	
df	1	
P value	0.1532	
P value summary	ns	
Are the survival curves sig different?	No	
Median survival		
T cell Delivery_FLT3L Vaccine	27	
T cell Delivery_GMCSF Vaccine	40	
Ratio (and its reciprocal)	0.675	1.481
95% CI of ratio	0.2514 to 1.812	0.5517 to 3.978
Hazard Ratio (Mantel-Haenszel)		
Ratio (and its reciprocal)	1.608	0.6218
95% CI of ratio	0.5584 to 4.632	0.2159 to 1.791
Hazard Ratio (logrank)		
Ratio (and its reciprocal)	1.54	0.6493
95% CI of ratio	0.5698 to 4.540	0.2203 to 1.755
**Figure 6h :Log-rank (Mantel-Cox) test**		
Comparison of Survival Curves	**NT vs T cell depot**	
Log-rank (Mantel-Cox) test		
Chi square	11.47	
df	1	
P value	0.0007	
P value summary	***	
Are the survival curves sig different?	Yes	
Gehan-Breslow-Wilcoxon test		
Chi square	10.33	
df	1	
P value	0.0013	
P value summary	**	
Are the survival curves sig different?	Yes	
Median survival		
Tumor Only Control	21	
T cells in Cryogel with IL2	32.5	
Ratio (and its reciprocal)	0.6462	1.548
95% CI of ratio	0.2343 to 1.782	0.5612 to 4.268

Hazard Ratio (Mantel-Haenszel)		
Ratio (and its reciprocal)	11.4	0.08776
95% CI of ratio	2.787 to 46.60	0.02146 to 0.3588
Hazard Ratio (logrank)		
Ratio (and its reciprocal)	4.072	0.2456
95% CI of ratio	3.353 to 38.73	0.02582 to 0.2983
Comparison of Survival Curves	**NT vs SIVETs**	
Log-rank (Mantel-Cox) test		
Chi square	18.06	
df	1	
P value	< 0.0001	
P value summary	****	
Are the survival curves sig different?	Yes	
Gehan-Breslow-Wilcoxon test		
Chi square	15.95	
df	1	
P value	< 0.0001	
P value summary	****	
Are the survival curves sig different?	Yes	
Median survival		
Tumor Only Control	21	
SIVET	67	
Ratio (and its reciprocal)	0.3134	3.19
95% CI of ratio	0.1176 to 0.8351	1.197 to 8.501
Hazard Ratio (Mantel-Haenszel)		
Ratio (and its reciprocal)	26.12	0.03829
95% CI of ratio	5.801 to 117.6	0.008503 to 0.1724
Hazard Ratio (logrank)		
Ratio (and its reciprocal)	5.494	0.182
95% CI of ratio	6.720 to 101.5	0.009850 to 0.1488
Comparison of Survival Curves	**NT vs Vax only**	
Log-rank (Mantel-Cox) test		
Chi square	14.44	
df	1	
P value	0.0001	
P value summary	***	
Are the survival curves sig different?	Yes	
Gehan-Breslow-Wilcoxon test		
Chi square	13.51	
df	1	
P value	0.0002	
P value summary	***	
Are the survival curves sig different?	Yes	
Median survival		
Tumor Only Control	21	
Vaccine Only	26.5	
Ratio (and its reciprocal)	0.7925	1.262
95% CI of ratio	0.3360 to 1.869	0.5350 to 2.976
Hazard Ratio (Mantel-Haenszel)		
Ratio (and its reciprocal)	15.78	0.06336
95% CI of ratio	3.804 to 65.49	0.01527 to 0.2629
Hazard Ratio (logrank)		
Ratio (and its reciprocal)	3.902	0.2562
95% CI of ratio	4.492 to 55.45	0.01803 to 0.2226
Comparison of Survival Curves	**T cell depot vs SIVETs**	
Log-rank (Mantel-Cox) test		
Chi square	1.864	
df	1	
P value	0.1721	
P value summary	ns	
Are the survival curves sig different?	No	
Gehan-Breslow-Wilcoxon test		
Chi square	0.9035	
df	1	
P value	0.3418	
P value summary	ns	
Are the survival curves sig different?	No	
Median survival		
T cells in Cryogel with IL2	32.5	
SIVET	67	
Ratio (and its reciprocal)	0.4851	2.062
95% CI of ratio	0.1759 to 1.338	0.7476 to 5.685
Hazard Ratio (Mantel-Haenszel)		
Ratio (and its reciprocal)	2.197	0.4552
95% CI of ratio	0.7098 to 6.800	0.1471 to 1.409
Hazard Ratio (logrank)		
Ratio (and its reciprocal)	1.959	0.5105
95% CI of ratio	0.7346 to 6.570	0.1522 to 1.361
Comparison of Survival Curves	**T cell depot vs Vax only**	
Log-rank (Mantel-Cox) test		
Chi square	0.4379	
df	1	

P value	0.5082					
P value summary	ns					
Are the survival curves sig different?	No					
Gehan-Breslow-Wilcoxon test						
Chi square	0.5684					
df	1					
P value	0.4509					
P value summary	ns					
Are the survival curves sig different?	No					
Median survival						
T cells in Cryogel with IL2	32.5					
Vaccine Only	26.5					
Ratio (and its reciprocal)	1.226	0.8154				
95% CI of ratio	0.5000 to 3.008	0.3325 to 2.000				
Hazard Ratio (Mantel-Haenszel)						
Ratio (and its reciprocal)	0.7318	1.367				
95% CI of ratio	0.2902 to 1.845	0.5419 to 3.446				
Hazard Ratio (logrank)						
Ratio (and its reciprocal)	0.7552	1.324				
95% CI of ratio	0.3096 to 1.729	0.5782 to 3.230				
Comparison of Survival Curves	**SIVETs vs Vax only**					
Log-rank (Mantel-Cox) test						
Chi square	6.09					
df	1					
P value	0.0136					
P value summary	*					
Are the survival curves sig different?	Yes					
Gehan-Breslow-Wilcoxon test						
Chi square	3.857					
df	1					
P value	0.0495					
P value summary	*					
Are the survival curves sig different?	Yes					
Median survival						
SIVET	67					
Vaccine Only	26.5					
Ratio (and its reciprocal)	2.528	0.3955				
95% CI of ratio	1.072 to 5.963	0.1677 to 0.9329				
Hazard Ratio (Mantel-Haenszel)						
Ratio (and its reciprocal)	0.3312	3.02				
95% CI of ratio	0.1377 to 0.7966	1.255 to 7.264				
Hazard Ratio (logrank)						
Ratio (and its reciprocal)	0.3746	2.67				
95% CI of ratio	0.1444 to 0.7596	1.317 to 6.926				
Comparison of Survival Curves	**SIVET FLT3L vs SIVET GMCSF**					
Log-rank (Mantel-Cox) test						
Chi square	0.4262					
df	1					
P value	0.5139					
P value summary	ns					
Are the survival curves sig different?	No					
Gehan-Breslow-Wilcoxon test						
Chi square	0.1127					
df	1					
P value	0.7371					
P value summary	ns					
Are the survival curves sig different?	No					
Median survival						
T cells in Cryogel with IL2_ A	Undefined					
T cells in Cryogel with IL2_ A	34					
Hazard Ratio (Mantel-Haenszel)						
Ratio (and its reciprocal)	0.6195	1.614				
95% CI of ratio	0.1472 to 2.608	0.3834 to 6.796				
Hazard Ratio (logrank)						
Ratio (and its reciprocal)	0.6339	1.578				
95% CI of ratio	0.1549 to 2.479	0.4035 to 6.458				

Supplementary Table 2- Statistical Information for Supplementary Figures

**Extended Fig 1a: Two-tailed one-way ANOVA with Geisser-Greenhouse correction**						
ANOVA summary						
F	51.64					
P value	< 0.0001					
P value summary	****					
Are differences among means statistically significant? (P < 0.05)	Yes					
R square	0.9538					
Brown-Forsythe test						
F (DFn	DFd)	0.4769 (4	10)			
P value	0.7523					
P value summary	ns					
Significantly different standard deviations? (P < 0.05)	No					
Bartlett's test						
Bartlett's statistic (corrected)						
P value						
P value summary						
Significantly different standard deviations? (P < 0.05)						
ANOVA table	SS	DF	MS	F (DFn	DFd)	P value
Treatment (between columns)	0.982	4	0.2455	F (4	10) = 51.64	P < 0.0001
Residual (within columns)	0.04755	10	0.004755			
Total	1.03	14				
Data summary						
Number of treatments (columns)	5					
Number of values (total)	15					
**Extended Fig 1b: Two-tailed one-way ANOVA with Geisser-Greenhouse correction**						
ANOVA summary						
F	4.894					
P value	0.019					
P value summary	*					
Are differences among means statistically significant? (P < 0.05)	Yes					
R square	0.6619					
Brown-Forsythe test						
F (DFn	DFd)	0.1417 (4	10)			
P value	0.9627					
P value summary	ns					
Significantly different standard deviations? (P < 0.05)	No					
Bartlett's test						
Bartlett's statistic (corrected)						
P value						
P value summary						
Significantly different standard deviations? (P < 0.05)						
ANOVA table	SS	DF	MS	F (DFn	DFd)	P value
Treatment (between columns)	0.003554	4	0.000888	F (4	10) = 4.894	P = 0.0190
Residual (within columns)	0.001815	10	0.000182			
Total	0.005369	14				
Data summary						
Number of treatments (columns)	5					
Number of values (total)	15					
**Extended Fig 1c: Two-tailed one-way ANOVA with Geisser-Greenhouse correction**						
ANOVA summary						
F	3.303					
P value	0.0571					
P value summary	ns					
Are differences among means statistically significant? (P < 0.05)	No					
R square	0.5692					
Brown-Forsythe test						
F (DFn	DFd)	0.6098 (4	10)			
P value	0.665					
P value summary	ns					
Significantly different standard deviations? (P < 0.05)	No					
Bartlett's test						
Bartlett's statistic (corrected)						

P value						
P value summary						
Significantly different standard deviations? (P < 0.05)						
ANOVA table	SS	DF	MS	F (DFn	DFd)	P value
Treatment (between columns)	0.0239	4	0.005974	F (4	10) = 3.303	P = 0.0571
Residual (within columns)	0.01809	10	0.001809			
Total	0.04198	14				
Data summary						
Number of treatments (columns)	5					
Number of values (total)	15					
**Extended Fig 1d: Two-tailed one-way ANOVA with Geisser-Greenhouse correction**						
ANOVA summary						
F	69.64					
P value	< 0.0001					
P value summary	****					
Are differences among means statistically significant? (P < 0.05)	Yes					
R square	0.9653					
Brown-Forsythe test						
F (DFn	DFd)	0.3605 (4	10)			
P value	0.8312					
P value summary	ns					
Significantly different standard deviations? (P < 0.05)	No					
Bartlett's test						
Bartlett's statistic (corrected)						
P value						
P value summary						
Significantly different standard deviations? (P < 0.05)						
ANOVA table	SS	DF	MS	F (DFn	DFd)	P value
Treatment (between columns)	0.5245	4	0.1311	F (4	10) = 69.64	P < 0.0001
Residual (within columns)	0.01883	10	0.001883			
Total	0.5434	14				
Data summary						
Number of treatments (columns)	5					
Number of values (total)	15					
**Extended Fig 2b: Two-way ANOVA with repeated measures**						
Alpha	0.05					
Source of Variation	% of total	P value	P value su	Significant?		
Interaction	13.38	< 0.0001	****	Yes		
Time	84.4	< 0.0001	****	Yes		
Column Factor	1.654	0.0007	***	Yes		
Subjects (matching)	0.07297	0.3313	ns	No		
ANOVA table	SS	DF	MS	F (DFn	DFd)	P value
Interaction	414.4	8	51.8	F (8	32) = 109	P < 0.0001
Time	2613	8	326.6	F (8	32) = 692	P < 0.0001
Column Factor	51.21	1	51.21	F (1	4) = 90.67	P = 0.0007
Subjects (matching)	2.259	4	0.5648	F (4	32) = 1.19	P = 0.3313
Residual	15.1	32	0.472			
Number of missing values	0					
**Extended Fig 2c: Two-way ANOVA with repeated measures**						
Alpha	0.05					
Source of Variation	% of total	P value	P value su	Significant?		
Interaction	57.14	< 0.0001	****	Yes		
Time	39.92	< 0.0001	****	Yes		
Column Factor	1.48	0.0084	**	Yes		
Subjects (matching)	0.2527	0.3145	ns	No		
ANOVA table	SS	DF	MS	F (DFn	DFd)	P value
Interaction	7.48E+06	6	1.25E+06	F (6	24) = 189	P < 0.0001
Time	5.23E+06	6	871497	F (6	24) = 132	P < 0.0001
Column Factor	193883	1	193883	F (1	4) = 23.43	P = 0.0084
Subjects (matching)	33101	4	8275	F (4	24) = 1.25	P = 0.3145
Residual	158141	24	6589			
Number of missing values	0					

**Extended Fig 2d: Two-way ANOVA with repeated measures**						
Alpha	0.05					
Source of Variation	% of total v	P value	P value su	Significant?		
Interaction	45.34	< 0.0001	****	Yes		
Time	45.99	< 0.0001	****	Yes		
Column Factor	7.608	0.0083	**	Yes		
Subjects (matching)	0.1279	0.4663	ns	No		
ANOVA table	SS	DF	MS	F (DFn	DFd)	P value
Interaction	2.33E+06	6	388131	F (6	12) = 96.1	P < 0.0001
Time	2.36E+06	6	393696	F (6	12) = 97.5	P < 0.0001
Column Factor	390833	1	390833	F (1	2) = 119.0	P = 0.0083
Subjects (matching)	6571	2	3285	F (2	12) = 0.81	P = 0.4663
Residual	48453	12	4038			
Number of missing values	0					
**Extended Fig 2f: Two-tailed unpaired t test with Welch's correction**						
Column B	Cytokine Loaded Depot					
vs.	vs.					
Column A	Blank Depot					
Unpaired t test with Welch's correction						
P value	< 0.0001					
P value summary	****					
Significantly different? (P < 0.05)	Yes					
One- or two-tailed P value?	Two-tailed					
Welch-corrected t	df	t=12.90 df=5.000				
How big is the difference?						
Mean ± SEM of column A	0.0 ± 0.0 N=5					
Mean ± SEM of column B	12.18 ± 0.9441 N=6					
Difference between means	12.18 ± 0.9441					
95% confidence interval	9.751 to 14.61					
R square	0.9708					
**Extended Fig 3c: Two-tailed unpaired t test with Welch's correction**						
Column B	Alginate-Collagen Hybrid Depot					
vs.	vs.					
Column A	Alginate Only Cryogel					
Unpaired t test with Welch's correction						
P value	0.0045					
P value summary	**					
Significantly different? (P < 0.05)	Yes					
One- or two-tailed P value?	Two-tailed					
Welch-corrected t	df	t=2.932 df=71.14				
How big is the difference?						
Mean ± SEM of column A	6.761 ± 0.2874 N=40					
Mean ± SEM of column B	8.196 ± 0.3963 N=40					
Difference between means	1.436 ± 0.4895					
95% confidence interval	0.4595 to 2.412					
R square	0.1078					
F test to compare variances						
F	DFn	Dfd	1.901	39	39	
P value	0.0482					
P value summary	*					
Significantly different? (P < 0.05)	Yes					
**Extended Fig 3d: Two-tailed unpaired t test with Welch's correction**						
Column B	Alginate-Collagen Hybrid Depot					
vs.	vs.					
Column A	Alginate Only Cryogel					
Unpaired t test with Welch's correction						
P value	0.0036					
P value summary	**					
Significantly different? (P < 0.05)	Yes					
One- or two-tailed P value?	Two-tailed					
Welch-corrected t	df	t=3.002 df=78.00				

How big is the difference?						
Mean ± SEM of column A	330.0 ± 20.86 N=40					
Mean ± SEM of column B	418.7 ± 20.94 N=40					
Difference between means	88.73 ± 29.56					
95% confidence interval	29.88 to 147.6					
R square	0.1036					
F test to compare variances						
F	DFn	Dfd	1.007	39	39	
P value	0.9824					
P value summary	ns					
Significantly different? (P < 0.05)	No					
**Extended Fig 4a: Two-tailed unpaired t test with Welch's correction**						
Column B	Depot					
vs.	vs.					
Column A	2D Culture					
Unpaired t test with Welch's correction						
P value	0.0124					
P value summary	*					
Significantly different? (P < 0.05)	Yes					
One- or two-tailed P value?	Two-tailed					
Welch-corrected t	df	t=3.633 df=5.585				
How big is the difference?						
Mean ± SEM of column A	0.1457 ± 0.004343 N=5					
Mean ± SEM of column B	0.1838 ± 0.009555 N=5					
Difference between means	0.03814 ± 0.01050					
95% confidence interval	0.01199 to 0.06429					
R square	0.7027					
F test to compare variances						
F	DFn	Dfd	4.84	4	4	
P value	0.1559					
P value summary	ns					
Significantly different? (P < 0.05)	No					
**Extended Fig 4b: Two-tailed unpaired t test with Welch's correction**						
Column B	Depot					
vs.	vs.					
Column A	2D Culture					
Unpaired t test with Welch's correction						
P value	0.0857					
P value summary	ns					
Significantly different? (P < 0.05)	No					
One- or two-tailed P value?	Two-tailed					
Welch-corrected t	df	t=2.045 df=6.143				
How big is the difference?						
Mean ± SEM of column A	0.1242 ± 0.03417 N=5					
Mean ± SEM of column B	0.0448 ± 0.01842 N=5					
Difference between means	-0.0794 ± 0.03882					
95% confidence interval	-0.1739 to 0.01505					
R square	0.4052					
F test to compare variances						
F	DFn	Dfd	3.443	4	4	
P value	0.2583					
P value summary	ns					
Significantly different? (P < 0.05)	No					
**Extended Fig 4c: Two-tailed unpaired t test with Welch's correction**						
Column B	Depot					
vs.	vs.					
Column A	2D Culture					
Unpaired t test with Welch's correction						
P value	0.0012					
P value summary	**					
Significantly different? (P < 0.05)	Yes					
One- or two-tailed P value?	Two-tailed					
Welch-corrected t	df	t=7.914 df=4.113				

How big is the difference?						
Mean ± SEM of column A	0.2347 ± 0.004607 N=5					
Mean ± SEM of column B	0.5433 ± 0.03872 N=5					
Difference between means	0.3086 ± 0.03900					
95% confidence interval	0.2015 to 0.4157					
R square	0.9384					
F test to compare variances						
F	DFn	Dfd	70.65	4	4	
P value	0.0012					
P value summary	**					
Significantly different? (P < 0.05)	Yes					
**Extended Fig 4d: Two-tailed unpaired t test with Welch's correction**						
Column B	Depot					
vs.	vs.					
Column A	2D Culture					
Unpaired t test with Welch's correction						
P value	0.127					
P value summary	ns					
Significantly different? (P < 0.05)	No					
One- or two-tailed P value?	Two-tailed					
Welch-corrected t	df	t=1.849 df=4.730				
How big is the difference?						
Mean ± SEM of column A	0.7540 ± 0.1801 N=5					
Mean ± SEM of column B	0.4060 ± 0.05464 N=5					
Difference between means	-0.3480 ± 0.1882					
95% confidence interval	-0.8401 to 0.1441					
R square	0.4196					
F test to compare variances						
F	DFn	Dfd	10.86	4	4	
P value	0.0403					
P value summary	*					
Significantly different? (P < 0.05)	Yes					
**Extended Fig 5: Two-tailed unpaired t test with Welch's correction**						
Column B	Cytokine Loaded Depot					
vs.	vs.					
Column A	Blank Depot					
Unpaired t test with Welch's correction						
P value	0.7627					
P value summary	ns					
Significantly different? (P < 0.05)	No					
One- or two-tailed P value?	Two-tailed					
Welch-corrected t	df	t=0.3132 df=7.478				
How big is the difference?						
Mean ± SEM of column A	0.2068 ± 0.08537 N=5					
Mean ± SEM of column B	0.2395 ± 0.06010 N=6					
Difference between means	0.0327 ± 0.1044					
95% confidence interval	-0.2110 to 0.2764					
R square	0.01295					
F test to compare variances						
F	DFn	Dfd	1.682	4	5	
P value	0.5779					
P value summary	ns					
Significantly different? (P < 0.05)	No					
**Extended Fig 6b: Two-way ANOVA with repeated measures**						
Two-way RM ANOVA	Matching: Stacked					
Alpha	0.05					
Source of Variation	% of total v	P value	P value sum	Significant?		
Interaction	26.29	< 0.0001	****	Yes		
Time	52.5	< 0.0001	****	Yes		
Column Factor	17.56	< 0.0001	****	Yes		
Subjects (matching)	1.265	0.0882	ns	No		
ANOVA table	SS	DF	MS	F (DFn	DFd)	P value

Interaction	5.33E+15	4	1.33E+15	F (4	24) = 66.08	P < 0.0001
Time	1.06E+16	4	2.66E+15	F (4	24) = 132.0	P < 0.0001
Column Factor	3.56E+15	1	3.56E+15	F (1	6) = 83.29	P < 0.0001
Subjects (matching)	2.56E+14	6	4.27E+13	F (6	24) = 2.120	P = 0.0882
Residual	4.84E+14	24	2.02E+13			
Number of missing values	0					
**Extended Fig 7: Two-tailed unpaired t test with Welch's correction**						
Column B	T cell Depot					
vs.	vs.					
Column A	No Treatment					
Unpaired t test with Welch's correction						
P value	0.0074					
P value summary	**					
Significantly different? (P < 0.05)	Yes					
One- or two-tailed P value?	Two-tailed					
Welch-corrected t	df	t=5.016 df=4.000				
How big is the difference?						
Mean ± SEM of column A	0.0 ± 0.0 N=5					
Mean ± SEM of column B	0.2520 ± 0.05024 N=5					
Difference between means	0.2520 ± 0.05024					
95% confidence interval	0.1125 to 0.3915					
R square	0.8628					
**Extended Fig 10b: Two-tailed one-way ANOVA with Geisser-Greenhouse correction**						
ANOVA summary						
F	6.304					
P value	0.0035					
P value summary	**					
Are differences among means statistically significant? (P < 0.05)	Yes					
R square	0.627					
Brown-Forsythe test						
F (DFn	DFd)	3.458 (4	15)			
P value	0.0342					
P value summary	*					
Significantly different standard deviations? (P < 0.05)	Yes					
Bartlett's test						
Bartlett's statistic (corrected)	6.368					
P value	0.1733					
P value summary	ns					
Significantly different standard deviations? (P < 0.05)	No					
ANOVA table	SS	DF	MS	F (DFn	DFd)	P value
Treatment (between columns)	3.50E+11	4	8.76E+10	F (4	15) = 6.304	P = 0.0035
Residual (within columns)	2.08E+11	15	1.39E+10			
Total	5.59E+11	19				
Data summary						
Number of treatments (columns)	5					
Number of values (total)	20					
**Extended Fig 14b: Two-tailed unpaired t test with Welch's correction**						
Comparison	**Naïve vs SIVET FLT3L**					
Unpaired t test with Welch's correction						
P value	0.225					
P value summary	ns					
Significantly different? (P < 0.05)	No					
One- or two-tailed P value?	Two-tailed					
Welch-corrected t	df	t=1.734 df=2.000				
How big is the difference?						
Mean ± SEM of column A	0.0145 ± 0.0005000 N=2					
Mean ± SEM of column B	0.1200 ± 0.06083 N=3					
Difference between means	0.1055 ± 0.06083					
95% confidence interval	-0.1562 to 0.3672					
R square	0.6006					
F test to compare variances						
F	DFn	Dfd				

P value						
P value summary						
Significantly different? (P < 0.05)						
Comparison	**Naïve vs SIVET GMCSF**					
Unpaired t test with Welch's correction						
P value	0.0374					
P value summary	*					
Significantly different? (P < 0.05)	Yes					
One- or two-tailed P value?	Two-tailed					
Welch-corrected t	df	t=3.574 df=3.001				
How big is the difference?						
Mean ± SEM of column A	0.0145 ± 0.0005000 N=2					
Mean ± SEM of column C	0.1373 ± 0.03434 N=4					
Difference between means	0.1228 ± 0.03434					
95% confidence interval	0.01348 to 0.2320					
R square	0.8098					
F test to compare variances						
F	DFn	Dfd				
P value						
P value summary						
Significantly different? (P < 0.05)						
**Extended Fig 14c: Two-tailed unpaired t test with Welch's correction**						
Comparison	**Naïve vs SIVET FLT3L**					
Unpaired t test with Welch's correction						
P value	0.1604					
P value summary	ns					
Significantly different? (P < 0.05)	No					
One- or two-tailed P value?	Two-tailed					
Welch-corrected t	df	t=2.126 df=2.117				
How big is the difference?						
Mean ± SEM of column A	0.01139 ± 0.003615 N=2					
Mean ± SEM of column B	0.05667 ± 0.02099 N=3					
Difference between means	0.04528 ± 0.02130					
95% confidence interval	-0.04171 to 0.1323					
R square	0.681					
F test to compare variances						
F	DFn	Dfd				
P value						
P value summary						
Significantly different? (P < 0.05)						
Comparison	**Naïve vs SIVET GMCSF**					
Unpaired t test with Welch's correction						
P value	0.0031					
P value summary	**					
Significantly different? (P < 0.05)	Yes					
One- or two-tailed P value?	Two-tailed					
Welch-corrected t	df	t=6.465 df=3.961				
How big is the difference?						
Mean ± SEM of column A	0.01139 ± 0.003615 N=2					
Mean ± SEM of column C	0.06275 ± 0.007075 N=4					
Difference between means	0.05137 ± 0.007945					
95% confidence interval	0.02922 to 0.07351					
R square	0.9134					
F test to compare variances						
F	DFn	Dfd				
P value						
P value summary						
Significantly different? (P < 0.05)						

Supplementary Table 3- Antibody Information			
	**Flow Cytometry Mouse Antibodies**		
**Antibody**	**Fluorophore**	**Catalog Number**	**Vendor**
CD3	PerCP/Cy5.5	100218	Biolegend
CD90.1	APC/Cy7	202520	Biolegend
CD4	BV711	100557	Biolegend
CD8	PE/Cy7	100722	Biolegend
CD62L	BV510	104441	Biolegend
CD44	FITC	103005	Biolegend
CD25	APC	102012	Biolegend
OX40	PE	119409	Biolegend
PD1	PE/Dazzle	109116	Biolegend
LAG3	BV421	125221	Biolegend
CD45	PerCP/Cy5.5	103132	Biolegend
CD11b	APC/Cy7	101226	Biolegend
CD11c	BV711	117349	Biolegend
F4/80	bV510	123135	Biolegend
CD3	PE/Cy7	100722	Biolegend
CD80	APC	104714	Biolegend
CD86	PE/Dazzle	105042	Biolegend
MHCII	FITC	107605	Biolegend
XCR1	BV421	148216	Biolegend
CD103	PE	156904	Biolegend
IFNG	FITC	554411	BD Biosciences
CD4	Alexa 594	100446	Biolegend
FOXP3	PE	126404	Biolegend
	**Immunofluorescence Mouse Antibodies**		
**Antibody**	**Fluorophore**	**Catalog Number**	**Vendor**
CD8	Alexa 488	100723	Biolegend
CD3	Alexa 647	100209	Biolegend
CD90.1	Alexa 647	202508	Biolegend
CD11b	Alexa 488	101217	Biolegend
CD11c	Alexa 594	117346	Biolegend
F4/80	Alexa 647	123122	Biolegend
