# Supplementary material for: Adoptive T cell transfer and host antigen-presenting cell recruitment with cryogel scaffolds promotes long-term protection against solid tumors
Source: Nat Commun. 2023 Jun 15;14:3546. doi: 10.1038/s41467-023-39330-7 (PMC10272124; doi:10.1038/s41467-023-39330-7)
Supplement: Supplementary file 4 — Reporting Summary [file 41467_2023_39330_MOESM4_ESM.pdf]

nature portfolio

Corresponding author(s): David J. Mooney

Last updated by author(s): 5/15/2023

Reporting Summary

Nature Portfolio wishes to improve the reproducibility of the work that we publish. This form provides structure for consistency and transparency in reporting. For further information on Nature Portfolio policies, see our Editorial Policies and the Editorial Policy Checklist.

Statistics

For all statistical analyses, confirm that the following items are present in the figure legend, table legend, main text, or Methods section.

n/a	Confirmed	
[ ]	[x]	The exact sample size (*n*) for each experimental group/condition, given as a discrete number and unit of measurement
[ ]	[x]	A statement on whether measurements were taken from distinct samples or whether the same sample was measured repeatedly
[ ]	[x]	The statistical test(s) used AND whether they are one- or two-sided<br>*Only common tests should be described solely by name; describe more complex techniques in the Methods section.*
[x]	[ ]	A description of all covariates tested
[ ]	[x]	A description of any assumptions or corrections, such as tests of normality and adjustment for multiple comparisons
[ ]	[x]	A full description of the statistical parameters including central tendency (e.g. means) or other basic estimates (e.g. regression coefficient) AND variation (e.g. standard deviation) or associated estimates of uncertainty (e.g. confidence intervals)
[ ]	[x]	For null hypothesis testing, the test statistic (e.g. *F*, *t*, *r*) with confidence intervals, effect sizes, degrees of freedom and *P* value noted<br>*Give P values as exact values whenever suitable.*
[x]	[ ]	For Bayesian analysis, information on the choice of priors and Markov chain Monte Carlo settings
[ ]	[x]	For hierarchical and complex designs, identification of the appropriate level for tests and full reporting of outcomes
[x]	[ ]	Estimates of effect sizes (e.g. Cohen's *d*, Pearson's *r*), indicating how they were calculated

Our web collection on statistics for biologists contains articles on many of the points above.

Software and code

Policy information about availability of computer code

Data collection	Softwares used for data acquisition are described in the Methods section of the manuscript. Flow cytometry data was collected on a BD Fortessa LSRII. SHG imaging was performed on a Leica SP5 X MP Inverted Confocal Microscope. SEM was performed using the Zeiss FESEM Ultra Plus
Data analysis	Flow cytometry analyses were performed using FlowJo version 10 or R version 4.0.5. SHG quantification was performed using Imaris. Dot plot and mouse survival analyses were performed using Graphpad Prism 6

For manuscripts utilizing custom algorithms or software that are central to the research but not yet described in published literature, software must be made available to editors and reviewers. We strongly encourage code deposition in a community repository (e.g. GitHub). See the Nature Portfolio guidelines for submitting code & software for further information.

Data

Policy information about availability of data

All manuscripts must include a data availability statement. This statement should provide the following information, where applicable:

- Accession codes, unique identifiers, or web links for publicly available datasets
- A description of any restrictions on data availability
- For clinical datasets or third party data, please ensure that the statement adheres to our policy

Requests for all other raw or analyzed data will be made available upon request.

Human research participants

Policy information about studies involving human research participants and Sex and Gender in Research.

Reporting on sex and gender	N/A
Population characteristics	N/A
Recruitment	N/A
Ethics oversight	N/A

Note that full information on the approval of the study protocol must also be provided in the manuscript.

Field-specific reporting

Please select the one below that is the best fit for your research. If you are not sure, read the appropriate sections before making your selection.

- [x] Life sciences
- [ ] Behavioural & social sciences
- [ ] Ecological, evolutionary & environmental sciences

For a reference copy of the document with all sections, see nature.com/documents/nr-reporting-summary-flat.pdf

Life sciences study design

All studies must disclose on these points even when the disclosure is negative.

Sample size	Sample sizes were chosen to yield statistical significance based on previous studies and historical literature
Data exclusions	No data were excluded
Replication	Replication of results included performing separate independent mouse therapeutic studies to determine reproducibility in results. Details are included in the figure legends
Randomization	Mice that have been inoculated with tumors were randomized prior to treatment to preserve similar tumor distribution
Blinding	Blinding was not performed for this study

Reporting for specific materials, systems and methods

We require information from authors about some types of materials, experimental systems and methods used in many studies. Here, indicate whether each material, system or method listed is relevant to your study. If you are not sure if a list item applies to your research, read the appropriate section before selecting a response.

Materials & experimental systems

n/a	Involved in the study	
	x	Antibodies
	x	Eukaryotic cell lines
x		Palaeontology and archaeology
	x	Animals and other organisms
x		Clinical data
x		Dual use research of concern

Methods

n/a	Involved in the study	
x		ChIP-seq
	x	Flow cytometry
x		MRI-based neuroimaging

Antibodies

Antibodies used

Antibody Fluorophore Catalog Number Vendor
CD3 PerCP/Cy5.5 100218 Biolegend
CD90.1 APC/Cy7 202520 Biolegend
CD4 BV711 100557 Biolegend
CD8 PE/Cy7 100722 Biolegend
CD62L BV510 104441 Biolegend
CD44 FITC 103005 Biolegend

CD25 APC 102012 Biolegend
OX40 PE 119409 Biolegend
PD1 PE/Dazzle 109116 Biolegend
LAG3 BV421 125221 Biolegend
CD45 PerCP/Cy5.5 103132 Biolegend
CD11b APC/Cy7 101226 Biolegend
CD11c BV711 117349 Biolegend
F4/80 bV510 123135 Biolegend
CD3 PE/Cy7 100722 Biolegend
CD80 APC 104714 Biolegend
CD86 PE/Dazzle 105042 Biolegend
MHCII FITC 107605 Biolegend
XCR1 BV421 148216 Biolegend
CD103 PE 156904 Biolegend
IFNG FITC 554411 BD Biosciences
CD4 Alexa 594 100446 Biolegend
CD8 Alexa 488 100723 Biolegend
CD3 Alexa 647 100209 Biolegend
CD90.1 Alexa 647 202508 Biolegend
CD11b Alexa 488 101217 Biolegend
CD11c Alexa 594 117346 Biolegend
F4/80 Alexa 647 123122 Biolegend
FOXP3 PE 126404 Biolegend

Validation

All antibodies are routinely tested by manufacturer

Eukaryotic cell lines

Policy information about cell lines and Sex and Gender in Research

Cell line source(s)

B16F10 was purchased from American Type Culture Collection (ATCC)
B16-cOVA was a kind gift from the Wucherpfennig lab

Authentication

B16F10 and B16-cOVA cell lines have been authenticated by ATCC and Wucherpfennig lab respectively. Cell lines were used at passage 7.

Mycoplasma contamination

No further mycoplasma contamination test have been performed on the cell lines beyond those that were performed by ATCC and the Wucherpfennig lab

Commonly misidentified lines
(See ICLAC register)

Name any commonly misidentified cell lines used in the study and provide a rationale for their use.

Animals and other research organisms

Policy information about studies involving animals; ARRIVE guidelines recommended for reporting animal research, and Sex and Gender in Research

Laboratory animals

Species Strain Age Vendor Catalog Number
Mouse C57BL/6J 7-9 weeks Jackson Labs #000664
Mouse B6.Cg-Thy1a/Cy Tg(TcraTcrb)8Rest/J 8 weeks Jackson Labs #005023
Mouse FVB-Tg(CAG-luc,-GFP)L2G85Chco/J 8 weeks Jackson Labs #008450
Mouse B6(Cg)-Tyrc-2J/J 8 weeks Jackson Labs #000058
Mouse C57BL/6-Tg(TcraTcrb)1100Mjb/J (OTI) 8 weeks Jackson Labs #003831

Wild animals

The study did not involve wild animals

Reporting on sex

Studies were performed using both female and male mice

Field-collected samples

Studies did not involve samples collected in the field

Ethics oversight

Animal studies were performed in accordance with the National Institutes of Health and the Harvard University Faculty of Arts and Sciences' Institutional Animal Care and Use Committee (IACUC) guidelines

Note that full information on the approval of the study protocol must also be provided in the manuscript.

Flow Cytometry

Plots

Confirm that:

- [x] The axis labels state the marker and fluorochrome used (e.g. CD4-FITC).
- [x] The axis scales are clearly visible. Include numbers along axes only for bottom left plot of group (a 'group' is an analysis of identical markers).
- [x] All plots are contour plots with outliers or pseudocolor plots.
- [x] A numerical value for number of cells or percentage (with statistics) is provided.

Methodology

Sample preparation

Tissue processing
Tumors: Tumors were excised into gentleMacs C tubes (Miltenyi #130-093-237) containing 150U/ml collagenase type IV (Thermo #17104019) and 0.1ug/ul DNAse 1 (Sigma #11284932001) in digestion media: RPMI 1640+10% FBS. Tumors were mechanically dissociated using the gentleMacs tissue dissociator (Miltenyi #130-093-235) program m_spleen_03, and incubated for 25 minutes at 37°C. Tumors were then mechanically dissociated for the second time using the same program and incubated for 15 extra minutes. The enzymatic reaction was quenched using MACS buffer: DPBS with 0.5% BSA and 2mM EDTA, and filtered through a 30um strainer (Miltenyi #130-098-458)
Lymph nodes: Brachial, axillary and inguinal lymph nodes were harvested and pooled before digestion. The same digestion protocol highlighted above was used to digest the lymph nodes.
Depots: Depots were harvested into gentleMacs C tubes containing 150U/ml collagenase type IV, 0.1ug/ul DNAse 1 and 2U/ml alginate lyase (Sigma #A1603). The same digestion protocol described above was used to digest depots.
Spleens: Harvested spleens were mechanically dissociated by using 1ml syringe plungers to mash the spleens through 30um strainers. Red blood cell lysis (BioLegend #420302) was performed on single cell suspension for 1 min before further downstream processing.
In vitro T cell restimulation
Lymph nodes and spleens were first digested as described above. To perform in vitro T cell stimulation, lymph node and spleen derived cells were incubated with a cocktail of 2ug/ml mgp100, M27 and M30 peptides in addition to 5e4 B16-F10 tumor cells. The broad approach to antigen stimulation was taken because the vaccine was antigen-free, and thus a broad repertoire of T cell clones was expected to be elicited by the vaccines. After 1.5hrs, 0.27ul of the GolgiStop protein transport inhibitor (BD #554724) was added to each well, after which the cells were incubated for 4 hrs. The cells were then processed for flow cytometry.
Flow cytometery
Surface staining: Cells were kept at 4°C throughout immunostaining. First, cells were stained with LIVE/DEAD™ Fixable Blue Dead Cell Stain (ThermoFisher Scientific #L23105) at 1000x dilution for 30mins in PBS, after which staining was quenched with flow cytometry staining (FACs) buffer (Invitrogen #00-4222-26). Cells were blocked with TruStain FcX Fc receptor blocking solution (BioLegend #101319) for 5 min and stained with surface protein antibodies for 20 min, after which the cells were washed 3x in FACs buffer. Flow cytometry acquisition was performed on a BD Fortessa LSRII. Single color compensation beads (Thermo #01-2222-41), was used for multi-parameter flow cytometry compensation. Gating was done based on fluorescence-minus-one (FMO) controls. Complete set of antibodies used for flow cytometry are listed in Supplementary Table 3.
Intracellular cytokine staining (ICS): ICS was performed after live/dead and surface staining, using the Cyto-Fast™ Fix/Perm Buffer Set (BioLegend #426803) according to the manufacturer's protocol. Briefly, cells were fixed in the Cyto-Fast™ Fix/Perm Buffer for 20 minutes at room temperature, washed twice in 1X Cyto-Fast™ Perm Wash solution and stained in 1X Cyto-Fast™ Perm Wash solution for 20 minutes at room temperature. After staining, cells were washed 3x in FACs buffer before acquisition on the BD Fortessa LSRII.

Instrument

BD Fortessa LSRII

Software

Facs DIVA was used to acquire flow cytometry data. FlowJo and R were used for analyses

Cell population abundance

No sorting was performed

Gating strategy

Fcs files exported from the BD Fortessa LSRII cytometer were imported into Flowjo, analyzed using the following hierarchy: SSC-A/FSC-A to gate for lymphocytes FSC-H/FSC-A to gate for single cells CD3/Live_Dead to gate for live T cells (or CD45/Live_Dead for total immune cells) CD4/CD8 to gate for CD4 or CD8 T cells. Further downstream gating is performed using FMO controls, or compensated single cell flow cytometry intensity values are exported as csv files for unsupervised analyses. Sample gating strategies are shown in Supplementary Figure 15

- [x] Tick this box to confirm that a figure exemplifying the gating strategy is provided in the Supplementary Information.
